# Supplementary material for: Home-based cardiac rehabilitation and physical activity in people with heart failure: a secondary analysis of the REACH-HF randomised controlled trials
Source: BMJ Open. 2023 Feb 9;13(2):e063284. doi: 10.1136/bmjopen-2022-063284 (PMC9923308; doi:10.1136/bmjopen-2022-063284)
Supplement: Supplementary data [file bmjopen-2022-063284supp001.pdf]

Supplementary tables

Supplementary table 1: Patient characteristics at baseline. Data are presented as N(%) unless otherwise stated.

	REACH-HF N=122	Control N=125
Mean (SD) Age (years)	70.5 (10.0)	71.3 (10.7)
Female Sex	40 (33)	30 (24)
Mean (SD) BMI (kg/m^2)	29.9 (6.6)	30.0 (5.9)
Employment status		
In employment/ Self-employed	15 (12)	16 (13)
Retired	98 (80)	101 (81)
Housework	0 (0)	1 (1)
Unemployed	7 (6)	3 (2)
Other	2 (2)	4 (3)
Ethnicity (white)	115 (94)	121 (97)
NYHA class		
NYHA I	24 (20)	16 (13)
NYHA II	71 (58)	76 (61)
NYHA III-IV	27 (22)	33 (26)
Time since HF diagnosis		
0 years	37 (30)	37 (30)
1 year	24 (20)	23 (18)
2 years	61 (50)	65 (52)
Cause of HF		
Ischaemic	52 (43)	63 (50)
Non-ischaemic	63 (52)	53 (43)
Not known/ classified	7 (5)	9 (7)
Mean (SD) LVEF (%)	38.4 (14.7)	38.1 (15.5)
Mean (SD) NT-proBNP (pg/ml)	1288.3 (1794.3)	1364.4 (1602.1)
Living alone	32 (26)	34 (27)
Living with partner	79 (65)	79 (63)
Living with child>18	7 (6)	10 (8)
Living with child<18	3 (2)	2 (2)
Living with parent	2 (2)	3 (2)
Smoking history		
Current smoker	7 (6)	7 (5)
Ex-smoker	67 (55)	72 (58)
Never smoked	48 (39)	46 (37)
Trial site		
Truro	27 (22)	29 (23)
Gwent	22 (18)	22 (18)
Birmingham	24 (20)	24 (19)
York	25 (20)	25 (20)
Dundee	24 (20)	25 (20)
Comorbidities		
Angina	32 (26)	33 (26)
Diabetes	31 (25)	28 (22)
MI	29 (24)	41 (33)
Hypertension	59 (48)	54 (43)
Osteoporosis	11 (9)	6 (5)

Stroke	14 (11)	15 (12)
Asthma	14 (11)	14 (11)
Chronic back pain	40 (33)	36 (29)
Chronic renal impairment	17 (14)	28 (22)
Arthritis	57 (47)	50 (40)
Atrial fibrillation	51 (42)	67 (54)
COPD	13 (11)	14 (11)
Depression	30 (25)	31 (25)
Total number of comorbidities	3 (2-5)	3 (2-5)
Medication		
Angiotensin II receptor antagonist	38 (31)	27 (22)
ACE inhibitor	71 (58)	84 (67)
Aldosterone antagonist	62 (51)	56 (45)
Anticoagulant	52 (43)	62 (50)
Beta blocker	99 (81)	95 (76)
Digoxin	23 (19)	16 (13)
Ivabradine	5 (4)	8 (6)
Loop diuretic	83 (68)	87 (70)
Nitrate	22 (18)	16 (13)
Thiazide diuretic	1 (1)	3 (2)
Type of HF (HFpEF)	24 (20)	25 (20)
Mean (SD) ISWT (peak distance, m)	241.9 (157.1)	219.1 (144.2)
Mean (SD) MLHFQ		
Overall	33.5 (24.5)	30.7 (23.0)
Physical	17.3 (11.8)	16.1 (11.6)
Emotional	7.7 (7.7)	7.1 (7.0)
Mean (SD) HADS		
Anxiety	5.3 (4.5)	5.9 (4.4)
Depression	4.8 (3.6)	4.8 (3.4)
Mean (SD) HeartQoL		
Global	1.8 (0.8)	1.8 (0.8)
Physical	1.6 (0.8)	1.6 (0.8)
Emotional	2.1 (0.9)	2.1 (0.9)
Mean (SD) EQ-5D-5L	0.7 (0.3)	0.7 (0.3)
Mean (SD) SCHFI		
Maintenance	56.0 (16.1)	53.0 (15.4)
Management	42.0 (24.7)	39.6 (20.4)
Confidence	61.9 (25.2)	63.6 (23.4)

REACH-HF: Rehabilitation enablement in chronic heart failure; SD: standard deviation; BMI: body mass index; NYHA: New York Heart Association; HF: heart failure; LVEF: left ventricular ejection fraction; NT-proBNP: N-terminal proB-type natriuretic peptide; MI: myocardial infection; COPD: chronic obstructive pulmonary disease; HFrEF: heart failure with reduced ejection fraction; HFpEF: heart failure with preserved ejection fraction; ISWT: incremental shuttle walk test; MLHFQ: Minnesota living with heart failure questionnaire; HADS: hospital anxiety and depression scale; SCHFI: self-care in heart failure index.

Supplementary table 2: Intervention effects on PA outcomes at post-intervention follow-up, bouted PA data

	Baseline		Post-intervention		Δ to post-intervention		Between group difference (mean, 95% CI) p-value
	REACH-HF (N=98) mean (sd)	Control (N=100) mean (sd)	REACH-HF (N=98) mean (sd)	Control (N=100) mean (sd)	REACH-HF (N=98) mean (sd)	Control (N=100) mean (sd)	
All days bouted							
MVPA (min/day)	37.90 (47.51)	44.96 (65.58)	39.64 (51.15)	46.96 (72.11)	1.74 (26.42)	2.00 (33.08)	-0.76 (-9.26 to 7.74) p=0.86
Light (min/day)	197.91 (103.22)	219.30 (108.05)	198.23 (105.26)	213.05 (114.25)	0.31 (70.48)	-6.25 (76.81)	0.98 (-19.09 to 21.06) p=0.92
Inactive (min/day)	1204.19 (133.00)	1175.74 (151.00)	1202.14 (140.26)	1179.99 (164.00)	-2.05 (76.55)	4.25 (92.03)	-2.40 (-26.20 to 21.40) p=0.84
Weekend days bouted							
MVPA (min/day)	39.01 (53.54)	37.03 (56.49)	36.62 (53.77)	43.99 (66.40)	-2.39 (33.09)	6.96 (50.56)	-9.12 (-20.82 to 2.57) p=0.13
Light (min/day)	194.02 (116.16)	198.50 (111.91)	182.82 (109.02)	192.50 (113.65)	-11.21 (88.87)	-6.00 (102.58)	-7.63 (-31.90 to 16.64) p=0.54
Inactive (min/day)	1206.97 (148.83)	1204.48 (145.62)	1220.56 (144.51)	1203.51 (155.21)	13.59 (96.46)	-0.97 (126.04)	15.84 (-13.92 to 45.59) p=0.30
Week days bouted							
MVPA (min/day)	37.46 (47.03)	48.13 (72.86)	40.85 (53.13)	48.15 (77.36)	3.39 (30.04)	0.01 (36.18)	2.26 (-7.16 to 11.67) p=0.64
Light (min/day)	199.47 (104.88)	227.63 (114.60)	204.39 (108.06)	221.27 (120.74)	4.92 (75.09)	-6.35 (81.07)	3.39 (-17.91 to 24.70) p=0.75
Inactive (min/day)	1203.07 (133.15)	1164.24 (161.83)	1194.76 (143.28)	1170.58 (174.37)	-8.31 (85.00)	6.34 (96.79)	-13.53 (-39.31 to 12.25) p=0.30)

REACH-HF: Rehabilitation enablement in chronic heart failure; SD: standard deviation; MVPA: moderate-to-vigorous physical activity; PA: physical activity

Supplementary table 3: Intervention effects at final follow-up, unbouted PA data

	Baseline		Final follow-up		Δ to final follow-up		Between group difference (mean, 95% CI) p-value
	REACH-HF (N=80) mean (SD)	Control (N=93) mean (SD)	REACH-HF (N=80) mean (SD)	Control (N=93) mean (SD)	REACH-HF(N=80) mean (SD)	Control (N=93) mean (SD)	
All days unbouted							
MVPA (min/day)	185.98 (86.30)	190.47 (96.18)	186.76 (97.24)	180.30 (97.22)	0.78 (55.62)	-10.17 (43.89)	9.86 (-4.96 to 24.69) p=0.19
Light (min/day)	195.39 (48.24)	194.29 (46.69)	193.36 (52.48)	189.97 (46.66)	-2.03 (33.65)	-4.32 (34.66)	2.35 (-7.65 to 12.35) p=0.64
Inactive (min/day)	1058.63 (108.27)	1055.24 (118.54)	1059.88 (121.89)	1069.73 (122.72)	1.25 (73.95)	14.49 (68.52)	-11.97 (-33.12 to 9.18) p=0.27
Weekend days unbouted							
MVPA (min/day)	183.15 (91.01)	175.14 (91.13)	173.76 (94.23)	169.86 (91.67)	-9.39 (62.73)	-5.28 (54.92)	-2.87 (-19.97 to 14.23) p=0.74
Light (min/day)	193.14 (54.28)	188.28 (49.54)	185.07 (55.34)	189.01 (50.88)	-8.07 (47.07)	0.73 (45.62)	-6.97 (-19.89 to 5.94) p=0.29
Inactive (min/day)	1063.71 (177.88)	1076.58 (120.30)	1081.17 (125.01)	1081.13 (118.25)	17.46 (92.05)	4.55 (83.95)	10.07 (-15.22 to 35.35) p=0.43
Week days unbouted							
MVPA (min/day)	187.11 (86.72)	196.60 (101.66)	191.96 (100.34)	184.47 (102.63)	4.85 (58.35)	-12.13 (45.46)*	15.18 (-0.32 to 30.67) p=0.06
Light (min/day)	196.29 (48.94)	196.70 (49.06)	196.68 (53.23)	190.36 (48.31)	0.39 (33.02)	-6.34 (37.88)	6.30 (-4.05 to 16.65) p=0.23
Inactive (min/day)	1056.60 (109.35)	1046.70 (123.47)	1051.36 (124.37)	1065.17 (129.8)	-5.24 (76.14)	18.47 (72.05)*	-21.25 (-43.24 to 0.75) p=0.06

REACH-HF: Rehabilitation enablement in chronic heart failure; SD: standard deviation; MVPA: moderate-to-vigorous physical activity; PA: physical activity

* p<0.05 REACH-HF group vs control

Supplementary table 4: Intervention effects at post-intervention follow-up, unbouted PA data

	Baseline		Post intervention		Δ to post-intervention		Between group difference (mean, 95% CI) p-value
	REACH-HF (N=98) mean (sd)	Control (N=100) mean (sd)	REACH-HF (N=98) mean (sd)	Control (N=100) mean (sd)	REACH-HF (N=98) mean (sd)	Control (N=100) mean (sd)	
All days Unbouted							
MVPA (min/day)	171.51 (79.28)	185.77 (88.79)	170.34 (83.87)	182.84 (95.19)	-1.17 (37.95)	-2.93 (44.70)	0.58 (-11.17 to 12.34) p=0.92
Light (min/day)	191.83 (47.69)	194.95 (46.02)	189.74 (46.55)	190.66 (41.19)	-2.09 (32.45)	-4.28 (32.49)	1.14 (-7.18 to 9.46) p=0.79
Inactive (min/day)	1076.66 (102.53)	1059.28 (110.43)	1079.92 (106.13)	1066.50 (114.70)	3.27 (60.16)	7.22 (68.83)	-0.65 (-18.56 to 17.26) p=0.94
Weekend days Unbouted							
MVPA (min/day)	169.77 (84.81)	170.92 (82.42)	160.49 (86.13)	171.80 (92.20)	-9.28 (45.26)	0.88 (63.94)	-10.48 (-25.78 to 4.81) p=0.18
Light (min/day)	189.50 (53.00)	187.11 (49.14)	181.25 (52.40)	181.35 (42.84)	-8.25 (46.60)	-5.76 (42.95)	-1.75 (-12.71 to 9.20) p=0.75
Inactive (min/day)	1080.73 (113.23)	1081.98 (111.68)	1098.26 (115.25)	1086.85 (114.35)	17.53 (79.97)	4.88 (93.05)	13.10 (-9.90 to 36.11) p=0.26
Week days Unbouted							
MVPA (min/day)	172.20 (79.63)	191.72 (94.89)	174.28 (85.32)	187.26 (99.35)	2.07 (42.27)	-4.46 (46.60)	4.29 (-8.27 to 16.86) p=0.50
Light (min/day)	192.77 (48.69)	198.08 (48.26)	193.14 (46.97)	194.39 (43.64)	0.37 (33.14)	-3.69 (34.36)	2.34 (-6.30 to 10.98) p=0.59
Inactive (min/day)	1075.03 (103.46)	1050.20 (115.91)	1072.59 (106.80)	1058.36 (119.56)	-2.44 (64.34)	8.15 (71.34)	-5.43 (-24.22 to 13.36) p=0.57

REACH-HF: Rehabilitation enablement in chronic heart failure; SD: standard deviation; MVPA: moderate-to-vigorous physical activity; PA: physical activity

Supplementary table 5: Intervention effects on proportion of patients meeting PA guidelines at final-follow up

	Baseline		Final follow-up		OR (95% CI) p-value
	REACH-HF (N=80) n (%)	Control (N=93) n (%)	REACH-HF (N=80) n (%)	Control (N=93) n (%)	
Bouted					
Proportion meeting guidelines	42 (53%)	45 (48%)	43 (54%)	39 (42%)	0.43 (0.16 to 1.14) p=0.09
Unbouted					
Proportion meeting guidelines	80 (100%)	93 (100%)	79 (99%)	93 (100%)	-

REACH-HF: Rehabilitation enablement in chronic heart failure; OR: odds ratio

Supplementary table 6: Intervention effects on proportion of patients meeting PA guidelines at post-intervention follow-up

	Baseline		Post-intervention		OR (95% CI) p-value
	REACH-HF (N=98) n (%)	Control (N=100) n (%)	REACH-HF (N=98) n (%)	Control (N=100) n (%)	
Bouted Proportion meeting guidelines	47, (48%)	47 (47%)	49 (50%)	47 (47%)	0.79 (0.34 to 1.84) p=0.59
Unbouted Proportion meeting guidelines	98 (100%)	100 (100%)	96, (98%)	100 (100%)	-

REACH-HF: Rehabilitation enablement in chronic heart failure; OR: odds ratio

Supplementary table 7: Univariate association with change in MVPA at final follow-up, controlling for trial stratifiers, group and baseline MVPA

Sociodemographic, exercise capacity and health status variables N=173 unless otherwise stated	Unstandardized beta coefficient (95% CI)	p-value
Age	-0.77 (-1.49 to -0.05)	0.04
Gender	1.19 (-13.50 to 15.89)	0.87
BMI	-0.46 (-1.68 to 0.75)	0.45
Employment status		
In employment/Self-employed	Comparison group	0.26
Retired	-17.13 (-38.46 to 4.19)	
Housework	-18.44 (-105.09 to 68.21)	
Unemployed	-39.19 (-82.28 to 4.41)	
Other	-38.71 (-84.28 to 6.86)	
Ethnicity (white vs other)	-20.33 (-56.51 to 15.86)	0.27
NYHA class		
NYHA I	Comparison group	0.10
NYHA II	-17.20 (-34.54 to 0.14)	
NYHA III-IV	-21.84 (-43.34 to -0.34)	
Time since HF diagnosis		
0 years	Comparison group	0.27
1 year	-11.12 (-30.01 to 7.76)	
2 years	-11.72 (-26.48 to 3.03)	
Cause of HF		
Ischaemic	Comparison group	0.44
Non-ischaemic	8.53 (-4.84 to 21.90)	
Not known/classified	0.91 (-30.46 to 32.29)	
LVEF (%) (N=137)	-0.52 (-1.57 to 0.52)	0.33
NT-proBNP (pg/ml)	-0.003 (-0.009 to 0.004)	0.42
Living alone	-3.06 (-18.40 to 12.28)	0.69
Living with partner	-3.06 (-18.40 to 12.28)	0.69
Living with child >18	25.98 (1.80 to 50.16)	0.04
Living with child <18	-1.16 (-50.64 to 48.33)	0.96
Living with parent	-45.62 (-94.86 to 3.62)	0.07
Smoking history		
Current smoker	Comparison group	0.61
Ex-smoker	16.14 (-16.57 to 48.84)	
Never smoked	16.28 (-17.23 to 49.80)	
Trial site		
Truro	Comparison group	0.17
Gwent	-9.09 (-27.90 to 9.72)	
Birmingham	15.20 (-5.94 to 36.35)	
York	-2.93 (-21.72 to 15.87)	
Dundee	-10.67 (-29.41 to 8.07)	

Comorbidities		
Angina	0.16 (-15.50 to 15.82)	0.98
Diabetes	-12.85 (-27.80 to 2.09)	0.09
MI	-5.75 (-20.12 to 8.62)	0.43
Hypertension	-1.92 (-15.17 to 11.33)	0.78
Osteoporosis	6.64 (-19.25 to 32.52)	0.61
Stroke	-7.21 (-27.07 to 12.64)	0.47
Asthma	4.16 (-18.08 to 26.41)	0.71
Chronic back pain	1.60 (-11.91 to 15.11)	0.82
Chronic renal impairment	-3.58 (-21.05 to 13.89)	0.69
Arthritis	9.77 (-3.38 to 22.91)	0.14
Atrial fibrillation	-10.54 (-23.55 to 2.47)	0.11
COPD	-15.62 (-35.03 to 3.79)	0.11
Depression	-2.0 (-17.05 to 13.06)	0.79
Total number of comorbidities	-1.29 (-4.45 to 1.87)	0.42
Total number of cardiorespiratory and metabolic comorbidities*	-4.56 (-9.47 to 0.35)	0.07
Total number of physical and musculoskeletal comorbidities†	3.85 (-3.25 to 10.95)	0.29
Medication		
Angiotensin II receptor antagonist	4.66 (-9.91 to 19.23)	0.53
ACE inhibitor	-0.59 (-14.16 to 12.98)	0.93
Aldosterone antagonist	4.13 (-9.52 to 17.78)	0.55
Anticoagulant	-7.62 (-20.70 to 5.47)	0.25
Beta blocker	-7.79 (-24.33 to 8.75)	0.35
Digoxin	-0.52 (-18.20 to 17.16)	0.95
Ivabradine	5.48 (-25.45 to 36.42)	0.73
Loop diuretic	-12.65 (-26.68 to 1.39)	0.08
Nitrate	-1.50 (-21.28 to 18.27)	0.88
Thiazide diuretic	-15.84 (-65.32 to 33.65)	0.53
Type of HF (HFrEF vs HFpEF)	collinearity	
ISWT (peak distance) (N=165) baseline	0.06 (0.01 to 0.12)	0.02
Overall ENMO	1.44 (-0.52 to 3.39)	0.15
MLHFQ	0.04 (-0.26 to 0.33)	0.81
Overall		
Physical	-0.14 (-0.74 to 0.47)	0.66
Emotional	0.47 (-0.43 to 1.36)	0.31
HADS		
Anxiety	1.83 (0.40 to 3.25)	0.01
Depression	-0.13 (-2.27 to 2.01)	0.91
HeartQoL		
Global	-2.09 (-11.01 to 6.82)	0.64
Physical	-0.66 (-9.02 to 7.69)	0.88
Emotional	-3.82 (-11.27 to 3.64)	0.31
EQ-5D-5L (N=172)	4.58 (-22.35 to 31.52)	0.74

SCHFI		
Maintenance	0.19 (-0.25 to 0.64)	0.39
Management (N=94)	-0.01 (-0.28 to 0.25)	0.92
Confidence	0.01 (-0.26 to 0.27)	0.96

BMI: body mass index; NYHA: New York Heart Association; HF: heart failure; LVEF: left ventricular ejection fraction; NT-proBNP: N-terminal proB-type natriuretic peptide; MI: myocardial infection; COPD: chronic obstructive pulmonary disease; HFrEF: heart failure with reduced ejection fraction; HFpEF: heart failure with preserved ejection fraction; ISWT: incremental shuttle walk test; ENMO: Euclidean norm minus one; MLHFQ: Minnesota living with heart failure questionnaire; HADS: hospital anxiety and depression scale; SCHFI: self-care in heart failure index.

Supplementary table 8: Univariate association with change in MVPA at post-intervention follow-up, controlling for trial stratifiers, group and baseline MVPA

Sociodemographic, exercise capacity and health status variables<br>N=198 unless otherwise stated	Unstandardized beta coefficient (95% CI)	p-value
Age	-0.39 (-0.85 to 0.08)	0.11
Gender	-5.74 (-15.85 to 4.37)	0.26
BMI	-0.19 (-1.00 to 0.63)	0.65
Employment status		
In employment/Self-employed	Comparison group	0.30
Retired	-10.83 (-25.08 to 3.41)	
Housework	-10.65 (-72.68 to 51.38)	
Unemployed	-29.16 (-56.92 to -1.40)	
Other	-3.58 (-32.96 to 25.81)	
Ethnicity (white vs other)	-1.91 (-27.60 to 23.77)	0.88
NYHA class		
NYHA I	Comparison group	0.17
NYHA II	-5.67 (-17.94 to 6.59)	
NYHA III-IV	-13.58 (-28.25 to 1.08)	
Time since HF diagnosis		
0 years	Comparison group	0.80
1 year	-3.15 (-16.06 to 9.76)	
2 years	-3.30 (-13.30 to 6.70)	
Cause of HF		
Ischaemic	Comparison group	0.78
Non-ischaemic	-2.90 (-11.93 to 6.12)	
Not known/classified	1.62 (-18.58 to 21.82)	
LVEF (%) (N=151)	-0.08 (-0.67 to 0.51)	0.79
NT-proBNP (pg/ml)	-0.0003 (-0.004 to 0.003)	0.87
Living alone	-5.25 (-15.76 to 5.25)	0.33
Living with partner	2.94 (-6.55 to 12.42)	0.61
Living with child >18	2.94 (-14.88 to 20.76)	0.33
Living with child <18	5.81 (-29.66 to 41.29)	0.32
Living with parent	25.68 (-1.81 to 53.16)	0.07
Smoking history		
Current smoker	Comparison group	0.21
Ex-smoker	6.47 (-14.09 to 27.04)	
Never smoked	-1.55 (-22.93 to 19.83)	
Trial site		
Truro	Comparison group	0.76
Gwent	4.19 (-8.66 to 17.03)	
Birmingham	5.20 (-8.71 to 19.12)	
York	-1.81 (-14.46 to 10.84)	
Dundee	-2.57 (-15.59 to 10.46)	

Comorbidities		
Angina	-1.72 (-11.93 to 8.48)	0.74
Diabetes	-12.67 (-22.52 to 2.81)	0.01
MI	3.43 (-6.40 to 12.26)	0.49
Hypertension	-2.67 (-11.57 to 6.23)	0.55
Osteoporosis	-10.64 (-29.90 to 8.61)	0.28
Stroke	-1.65 (-15.44 to 12.14)	0.81
Asthma	8.83 (-5.38 to 23.04)	0.22
Chronic back pain	-8.46 (-17.87 to 0.94)	0.08
Chronic renal impairment	0.01 (-0.05 to 0.07)	0.68
Arthritis	-2.62 (-11.67 to 6.43)	0.57
Atrial fibrillation	-2.47 (-11.36 to 6.42)	0.58
COPD	-4.18 (-18.58 to 10.21)	0.57
Depression	-3.33 (-13.99 to 7.32)	0.54
Total number of comorbidities	-1.05 (-3.13 to 1.04)	0.32
Total number of cardiorespiratory and metabolic comorbidities*	-1.77 (-5.04 to 1.51)	0.29
Total number of physical and musculoskeletal comorbidities†	-3.33 (-8.37 to 1.71)	0.19
Medication		
Angiotensin II receptor antagonist	-0.99 (-10.74 to 8.76)	0.84
ACE inhibitor	5.54 (-3.47 to 14.56)	0.23
Aldosterone antagonist	3.87 (-5.29 to 13. 03)	0.83
Anticoagulant	-8.31 (-17.13 to 0.51)	0.07
Beta blocker	-3.34 (-14.57 to 7.89)	0.56
Digoxin	-2.71 (-14.63 to 9.21)	0.65
Ivabradine	12.82 (-5.30 to 30.94)	0.16
Loop diuretic	1.37 (-8.41 to 11.15)	0.78
Nitrate	-9.24 (-24.63 to 3.15)	0.14
Thiazide diuretic	-13.77 (-49.04 to 21.50)	0.44
Type of HF (HFrEF vs HFpEF)	-2.57 (-15.59 to 10.46)	0.70
ISWT (peak distance) (N=188) baseline	0.06 (0.02 to 0.09)	0.001
Overall ENMO	1.12 (-0.23 to 2.47)	0.10
MLHFQ	-0.12 (-0.32 to 0.07)	0.21
Overall		
Physical	-0.26 (-0.65 to 0.13)	0.20
Emotional	-.025 (-0.86 to 0.36)	0.42
HADS		
Anxiety	0.30 (-0.71 to 1.32)	0.56
Depression	-0.01 (-1.39 to 1.37)	0.99
HeartQoL		
Global	4.36 (-1.58 to 10.30)	0.15
Physical	4.52 (-0.97 to 10.00)	0.11
Emotional	1.56 (-3.62 to 6.75)	0.55
EQ-5D-5L (N=196)	7.54 (-10.96 to 26.03)	0.42

SCHFI		
Maintenance	-0.32 (-0.60 to -0.04)	0.03
Management (N=106)	0.06 (-0.22 to 0.34)	0.69
Confidence	-0.07 (-0.25 to 0.11)	0.43

BMI: body mass index; NYHA: New York Heart Association; HF: heart failure; LVEF: left ventricular ejection fraction; NT-proBNP: N-terminal proB-type natriuretic peptide; MI: myocardial infection; COPD: chronic obstructive pulmonary disease; HFrEF: heart failure with reduced ejection fraction; HFpEF: heart failure with preserved ejection fraction; ISWT: incremental shuttle walk test; ENMO: Euclidean norm minus one; MLHFQ: Minnesota living with heart failure questionnaire; HADS: hospital anxiety and depression scale; SCHFI: self-care in heart failure index.

Supplementary table 9: Comparison of multivariable models to predict change in minutes/day MVPA at post-intervention follow up.

Multivariable model	Variables included in model (p<0.05)	Unstandardized beta coefficient (95% CI)	t-statistic	Variable P-value	Model Adjusted R^2 (p-value)
1. Socio-demographic	Group	0.48 (-6.24 to 7.22)	0.14	0.89	0.10 (<0.001)
	Baseline MVPA	-0.12 (-0.18 to -0.06)	-3.81	<0.001	
	Centre	0.18 (-2.12 to 2.54)	0.15	0.88	
	BNP <>2000	-3.93 (-12.96 to 5.11)	-0.86	0.39	
	Live with parent	34.98 (13.41 to 56.55)	3.20	0.002	
	Diabetes	-12.17 (-20.08 to -4.27)	-3.04	0.003	
	constant	7.13 (-2.08 to 16.34)	1.53	0.13	
2. Exercise capacity and health status	Group	0.18 (-6.0 to 6.34)	0.06	0.95	0.10 (<0.001)
	Baseline MVPA	-0.15 (-0.21 to -0.09)	-4.73	<0.001	
	Centre	0.54 (-1.61 to 2.68)	0.49	0.62	
	BNP 2000	-2.03 (-10.29 to 6.23)	-0.49	0.63	
	ISWT peak	0.04 (0.02 to 0.07)	3.52	0.001	
	constant	-6.39 (-16.77 to 3.99)	-1.21	0.23	
3. Socio-demographic, exercise capacity and health status*	Group	2.02 (-4.8 to 8.83)	0.58	0.56	0.14 (<0.001)
	Baseline MVPA	-0.16 (-0.23 to -0.09)	-4.55	<0.001	
	Centre	0.80 (-1.59 to 3.20)	0.66	0.51	
	BNP 2000	-3.67 (-12.83 to 5.48)	-0.79	0.43	
	Live with parent	37.47 (13.69 to 61.24)	3.11	0.002	
	Diabetes	-11.99 (-19.98 to -4.01)	-2.96	0.003	
	ISWT peak	0.04 (0.01 to 0.06)	2.82	0.005	
	constant	-2.75 (-14.31 to 8.82)	-0.47	0.64	

MVPA: moderate-to-vigorous physical activity; BNP 2000: NT-proBNP above or below 2000 pg/ml; ISWT: incremental shuttle walk test; HADS: hospital anxiety and depression score
* all variables p<0.05 from multivariate models 1 and 2
