# Supplementary material for: Home-based cardiac rehabilitation and physical activity in people with heart failure: a secondary analysis of the REACH-HF randomised controlled trials
Source: BMJ Open. 2023 Feb 9;13(2):e063284. doi: 10.1136/bmjopen-2022-063284 (PMC9923308; doi:10.1136/bmjopen-2022-063284)
Supplement: Author's manuscript [file bmjopen-2022-063284.draft_revisions.pdf]

BMJ Open

BMJ Open is committed to open peer review. As part of this commitment we make the peer review history of every article we publish publicly available.

When an article is published we post the peer reviewers' comments and the authors' responses online. We also post the versions of the paper that were used during peer review. These are the versions that the peer review comments apply to.

The versions of the paper that follow are the versions that were submitted during the peer review process. They are not the versions of record or the final published versions. They should not be cited or distributed as the published version of this manuscript.

BMJ Open is an open access journal and the full, final, typeset and author-corrected version of record of the manuscript is available on our site with no access controls, subscription charges or pay-per-view fees (http://bmjopen.bmj.com).

If you have any questions on BMJ Open's open peer review process please email info.bmjopen@bmj.com

BMJ Open

Home-based cardiac rehabilitation and physical activity in people with heart failure: a secondary analysis of the REACH-HF randomised controlled trials

Journal:	*BMJ Open*
Manuscript ID	bmjopen-2022-063284
Article Type:	Original research
Date Submitted by the Author:	28-Mar-2022
Complete List of Authors:	Dibben, Grace; University of Glasgow, MRC/CSO Social and Public Health Sciences Unit; University of Exeter, College of Medicine and Health<br>Hillsdon, Melvyn; University of Exeter, Department of Sport and Health Sciences<br>Dalal, Hasnain; Royal Cornwall Hospitals NHS Trust, Research, Development and Innovation; University of Exeter Medical School, Primary Care<br>Tang, Lars; Slagelse Hospital, The Research Unit PROgrez, Department of Physiotherapy and Occupational Therapy; University of Southern Denmark, The Department of Regional Health Research<br>Doherty, Patrick Joseph; University of York, Department of Health Sciences<br>Taylor, Rod; University of Glasgow, MRC/CSO Social and Public Health Sciences Unit; University of Exeter, College of Medicine and Health
Keywords:	Heart failure < CARDIOLOGY, REHABILITATION MEDICINE, SPORTS MEDICINE

SCHOLARONE™
Manuscripts

I, the Submitting Author has the right to grant and does grant on behalf of all authors of the Work (as defined in the below author licence), an exclusive licence and/or a non-exclusive licence for contributions from authors who are: i) UK Crown employees; ii) where BMJ has agreed a CC-BY licence shall apply, and/or iii) in accordance with the terms applicable for US Federal Government officers or employees acting as part of their official duties; on a worldwide, perpetual, irrevocable, royalty-free basis to BMJ Publishing Group Ltd ("BMJ") its licensees and where the relevant Journal is co-owned by BMJ to the co-owners of the Journal, to publish the Work in this journal and any other BMJ products and to exploit all rights, as set out in our licence.

The Submitting Author accepts and understands that any supply made under these terms is made by BMJ to the Submitting Author unless you are acting as an employee on behalf of your employer or a postgraduate student of an affiliated institution which is paying any applicable article publishing charge ("APC") for Open Access articles. Where the Submitting Author wishes to make the Work available on an Open Access basis (and intends to pay the relevant APC), the terms of reuse of such Open Access shall be governed by a Creative Commons licence – details of these licences and which Creative Commons licence will apply to this Work are set out in our licence referred to above.

Other than as permitted in any relevant BMJ Author's Self Archiving Policies, I confirm this Work has not been accepted for publication elsewhere, is not being considered for publication elsewhere and does not duplicate material already published. I confirm all authors consent to publication of this Work and authorise the granting of this licence.

For peer review only

Home-based cardiac rehabilitation and physical activity in people with heart failure: a secondary analysis of the REACH-HF randomised controlled trials

Grace O Dibben[a,b], Melvyn Hillsdon[c], Hasnain M Dalal[b,d], Lars Hermann Tang[e,f], Patrick Doherty[g], Rod S Taylor[a,b]

Author affiliations:

[a] Institute of Health and Wellbeing, University of Glasgow, Glasgow, UK.

[b] College of Medicine and Health, University of Exeter, Exeter, UK.

[c] Department of Sport and Health Sciences, University of Exeter, Exeter, UK.

[d] Royal Cornwall Hospitals NHS Trust, Truro, UK

[e] The Research Unit PROgrez, Department of Physiotherapy and Occupational Therapy, Næstved-Slagelse-Ringsted Hospitals, Denmark.

[f] The Department of Regional Health Research, University of Southern Denmark, Denmark.

[g] Department of Health Sciences, University of York, York, UK.

Corresponding author:

Grace Dibben, MRC/CSO Social and Public Health Sciences Unit, University of Glasgow, Berkeley Square, 99 Berkeley Street, Glasgow, G3 7HR

Grace.Dibben@glasgow.ac.uk

Word count: 3309

For peer review only - http://bmjopen.bmj.com/site/about/guidelines.xhtml

Abstract

Objective: To quantify the impact of a home-based cardiac rehabilitation intervention (REACH-HF) on objectively assessed physical activity (PA) of heart failure (HF) patients and explore the extent by which patient characteristics are associated with a change in PA.

Methods: In this secondary analysis, objective PA data were pooled from two randomised controlled trials (RCTs) of REACH-HF plus usual care versus usual care alone (control). PA was assessed over 7 days via GENEActiv triaxial accelerometer at baseline (pre-randomisation), post-intervention (4 months) and final follow-up (6 to 12 months). Using HF patient specific intensity thresholds, intervention effects (REACH-HF vs control) on average min/day PA (inactivity, light PA and moderate-to-vigorous PA [MVPA]) over all days, week days and weekend days were examined using linear regression analysis. Multivariable regression was used to explore associations between baseline patient characteristics and change in PA.

Results: 247 HF patients (mean age 70.9±10.3 years; 28% female) were included in the analysis. Although there was no difference between REACH-HF and control groups in 7-day PA levels post-intervention or at final follow-up, there was evidence of an increase in weekday MVPA (10.9 mins/day, 95%CI: -2.94 to 24.69), light PA (26.9 mins/day, 95%CI: -0.05 to 53.8) and decreased inactivity (-38.31 mins/day, 95%CI: -72.1 to -4.5) in favour of REACH-HF. Baseline factors associated with an increase in PA from baseline to final follow-up were reduced MVPA, increased incremental shuttle walk test distance, increased HADS anxiety score, and living with a child >18 years ($p<0.05$).

Conclusions: Whilst participation in the REACH-HF home-based CR intervention did not increase overall weekly activity, patient's behaviour patterns appeared to change with increased weekday PA levels, and reduced inactivity. Baseline PA levels were highly

For peer review only - http://bmjopen.bmj.com/site/about/guidelines.xhtml

predictive of PA change. Clinicians and researchers should focus on robust behavioural changes, improving overall levels of objectively assessed PA of people with HF.

Trial registrations: ISRCTN78539530 and ISRCTN86234930.

Abstract word count: 300

Strengths and limitations of this study

- The use of population specific intensity thresholds to derive PA intensity provides a more reliable and robust estimate of PA levels of HF patients.
- The use of rigorous data processing and analysis methods to look beyond a single PA metric (i.e. average weekly MVPA) revealed interesting patterns in the data, however this increase in granularity causes a reduction in statistical power and future studies should consider this in sample size calculations
- As this was an exploratory study, there may be an increased risk of Type 1 errors with multiple repeated tests, and some frequency counts of variables included in multivariable models were low.

For peer review only

Introduction

Physical activity (PA) has numerous health benefits for heart failure (HF) patients including reduced HF mortality and HF hospitalisation, and improved quality of life.[1-3] Current PA guidelines recommend 150 mins/week moderate-to-vigorous PA (MVPA) or the equivalent of 30 mins/day on 5 or more days/week.[4-5]

Traditionally, cardiac rehabilitation (CR) interventions have prioritised increasing exercise capacity rather than PA behaviour, and evidence that CR increases PA toward recommended levels is lacking.[6] To date, only a small number of studies have assessed the impact of CR on PA levels in HF patients, mainly using self-report measures that are known to be prone to over-reporting.[7] HF specific accelerometer intensity thresholds for categorising PA intensity from raw accelerometer data have recently been developed, taking into account the lower resting metabolic rate and requirement for greater energy expenditure during PA in people with HF.[8] Furthermore, although some studies have shown that PA levels are associated with a number of factors including age, body mass index (BMI), exercise capacity and disease severity in HF patients, these have been limited by cross-sectional design that do not measure within-person PA change.[9-11]

Due to the wide range of health benefits associated with increased PA in HF patients, further studies, using improved, objective and population specific PA assessment techniques are needed to understand and clarify the impact of CR on PA levels and the relationships between PA and patient level characteristics i.e., sociodemographics, exercise capacity and quality of life. Therefore the primary aim of this study was to assess the impact of a home-based CR programme (Rehabilitation Enablement in Chronic Heart Failure (REACH-HF)[12-15] on objectively measured PA using HF specific accelerometer thresholds for

For peer review only - http://bmjopen.bmj.com/site/about/guidelines.xhtml

estimating intensity. In addition, we explored the patient level characteristics associated with a change in PA level.

Methods

Patient and Public Involvement

A key element of the REACH-HF intervention development process was the involvement of a local patient and public involvement group consisting of people with HF and caregivers of people with HF.

Study design

This secondary analysis used data pooled from two randomised controlled trials (RCTs) that randomised HF patients 1:1 to a home-based CR intervention plus usual care (REACH-HF group) or to usual care alone (control group), stratified by site and N-terminal Brain Natriuretic Peptide (NT-proBNP) (ISRCTN78539530 and ISRCTN86234930). The first was a pilot RCT of the REACH-HF intervention for patients with HF with preserved ejection fraction (HFpEF, left ventricular ejection fraction [LVEF] >45%), and the second was a multicentre RCT of the REACH-HF intervention for patients with HF with reduced ejection fraction (HFrEF, LVEF <45%). The primary outcome was change in disease-specific health related quality of life (HRQoL) with accelerometer assessed PA collected as a secondary outcome. Full details of these trials are presented elsewhere.[12-15]

In summary, REACH-HF is a theory-based, comprehensive self-management programme consisting of four core elements; (1) a HF self-help manual with a choice of two structured exercise programmes; a chair-based exercise DVD, and a progressive walking training programme (patients advised to exercise ≥3 times per week and gradually building in time/distance/walking pace); (2) a patient 'progress tracker' booklet designed to facilitate learning and record symptoms and other actions related to health care; (3) a 'family and

For peer review only - http://bmjopen.bmj.com/site/about/guidelines.xhtml

friends resource' manual for caregivers to increase their understanding of HF and their own physical and mental wellbeing; and (4) facilitation by specially trained cardiac nurses,physiotherapists, or exercise therapists to individually tailor the intervention. Participating patients and caregivers worked through the REACH-HF manual for 12 weeks. Participants in both the pilot RCT and full RCT received the same programme.

This study complies with the Declaration of Helsinki. Ethical approval was granted for the two trials by East of Scotland Research Ethics Service (15/ES/0036) and the North West Lancaster Research Ethics Committee (14/NW/1351).

Participants

A total of 266 HF patients (216 HFrEF, 50 HFpEF) were recruited and completed the baseline visit between January 2015 and February 2016, from primary and secondary care settings in five UK centres (Birmingham, Cornwall, Dundee, Gwent and York). Full inclusion and exclusion criteria are published elsewhere.[16-17] In summary, eligible participants were aged ≥18 years with a confirmed diagnosis of HF on echocardiography, radionuclide ventriculography or angiography within the last 6 months. All participants provided written informed consent.

Data collection

PA data were collected via accelerometry on three occasions: at baseline (pre-randomisation), post-intervention (4 months) and final follow-up (6-12 months). The final follow-up visit varied between the two trials (6 months for HFpEF patients, and 12 months for HFrEF patients) and have therefore been combined for this study.

During the baseline visit, the following data were collected: past medical history (i.e. comorbidities, New York Heart Association (NYHA) class, concomitant medication), sociodemographic information (i.e. age, ethnicity, employment status, smoking status), NT-

For peer review only - http://bmjopen.bmj.com/site/about/guidelines.xhtml

For peer review only

proBNP measurement via blood sample, exercise tolerance via incremental shuttle walk test (ISWT), and health outcome questionnaires i.e. disease-specific HRQoL using the Minnesota Living with Heart Failure Questionnaire (MLHFQ), and the Health Related Quality of Life (HeartQoL) questionnaire; psychological wellbeing using the Hospital Anxiety and Depression Scale (HADS) questionnaire; generic HRQoL using the EQ-5D-5L questionnaire; and Self-care of HF Index questionnaire (SCHFI).

Physical activity – accelerometry

At the clinic visits, participants were provided with and instructed to wear a GENEActiv triaxial accelerometer (GENEActiv, Activinsights, Kimbolton, Cambridge, UK) for 24-consecutive hours for 7 days. Accelerometers were returned to the clinical trials unit using postage-paid envelopes. Data were downloaded using GENEActiv PC software (version 3.2; Activinsights, Kimbolton, Cambridge, UK) and processed in R (R Core Team, Vienna, Austria) using the GGIR software package (version 1.5-18, http://cran.r-project.org). Initial processing included autocalibration, and detection of abnormally high values and non-wear.[18-19] Data were averaged over 5 second epochs and Euclidean Norm Minus One (ENMO) was used to quantify the acceleration related to movement registered and expressed in milligravity units (mg) using the following formula.[20]

$$\sqrt{x^2 + y^2 + z^2} - 1000mg$$

Non-wear was determined over 60-minute windows using 15 minute increments, and was apparent when 2 of the 3 axes had a data range <50 mg and a SD <13 mg.[21] To be included in analysis participants were required to have ≥16 hours per day and ≥7 days of wear. The first consecutive seven days that met the criteria were used for analysis.

Once the raw accelerometer data was processed, HF specific accelerometer intensity thresholds (inactivity (i.e. <1.5 metabolic equivalents [METs]: 16.7mg (left wrist), 18.6mg

For peer review only - http://bmjopen.bmj.com/site/about/guidelines.xhtml

(right wrist), MVPA (i.e. ≥3.0METs): 43.6mg (left wrist), 45.5mg (right wrist)) were applied to calculate the average minutes per day spent inactive, in light PA and MVPA, over all days, weekend days and weekdays. These thresholds were established by a recent accelerometer calibration study in 21 HF patients.[8] Average weekly MVPA (≥150 minutes per week) was used to calculate the proportion of patients meeting current PA guidelines. For the primary analysis these metrics were calculated using bouted data, i.e., sustained periods of 10 minutes or more where accelerometer data lies above the intensity threshold (with a 20% allowance for values to fall outside the threshold). For secondary analyses, these metrics were calculated using unbouted data, i.e. allowing PA to be accumulated in bouts of any duration.

Statistical analysis

Descriptive statistics were calculated as means and standard deviations or counts and percentages unless otherwise stated.

Data from final follow-up were used for primary analysis in line with the primary end-points of the two trials.[16-17] The intervention effects (i.e. REACH-HF vs control) on average min/day PA (inactivity, light PA and MVPA) over all days, week days and weekend days were examined using linear regression analysis, adjusting for baseline PA (inactivity, light PA or MVPA respectively), treatment group and trial stratification variables (NT-proBNP, centre). Similarly, intervention effects on the proportion of patients meeting PA guidelines was examined using logistic regression. These analyses were repeated at post-intervention follow-up and using unbouted PA data for secondary analyses.

Linear regression was used to investigate the baseline sociodemographic (e.g. age, sex, ethnicity), medical history (e.g. NYHA class, medication), ISWT and health status variables (e.g. HRQoL) associated with change in MVPA, adjusting for baseline MVPA treatment group and trial stratification variables (NT-proBNP, centre).

For peer review only - http://bmjopen.bmj.com/site/about/guidelines.xhtml

Variables with statistical evidence of univariate association with change in MVPA ($p<0.15$) were selected for entry into a series of multivariable regression models to establish which variables were independently and most strongly associated with change in PA at final follow-up and post-intervention, mutually adjusting for trial stratification variables, baseline MVPA and treatment group. Model 1 – sociodemographic and medical history variables only, model 2 – exercise capacity and health status variables only, and model 3 – sociodemographic, medical history, exercise capacity and health status variables identified as significant ($p<0.05$) predictors in models 1 and 2. Checks and diagnostics were performed for model assumptions, residuals, multicollinearity (variance inflation factor) and influential observations (Cook's distance). AIC and R^2 values were used to inform model comparison and selection.

Statistical analyses were performed using Stata (V.15.0; StataCorp, College Station, Texas, USA).

Results

247 patients had accelerometer data at baseline which met the criteria for inclusion in analysis. Post-intervention, 198 patients were included, and at final follow-up 173 patients were included in the analysis (Figure 1). Patients were predominantly male (72%), had a mean age of 70.9 ± 10.4 years, and 60% were NYHA class II (table 1), full baseline characteristics are described in supplementary table 1. PA levels did not differ between HFrEF and HFpEF patients at any time point (data not shown). At baseline, the average daily MVPA in REACH-HF group was 43.6 mins with 53% participants meeting weekly PA recommendations, and in the control group was 49.7 mins per day, with 48% participants meeting weekly PA recommendations.

Intervention analysis

For peer review only - http://bmjopen.bmj.com/site/about/guidelines.xhtml

The average change in daily MVPA (over all 7 days) at final follow-up in the REACH-HF group was 4.0 mins/day, but varied greatly (minimum -91.2, maximum 291.7, SD 51.5 min/day). The control group average MVPA change at final follow-up was -5.1 mins/day (min -135.7, max 173.2, SD 34.2 min/day). No between group differences were seen in PA changes over all days or weekend days (table 2). However, an increase in at least light intensity, bouted PA on weekdays and a decrease in weekday inactivity (mins/day) was seen in the REACH-HF group with the reverse found in the control group (between group differences in favour of REACH-HF: light PA: 26.87 (95% CI -0.05 to 53.78), p=0.05, inactivity: -38.31 (95% CI -72.13 to -4.5), p=0.03).

In terms of prevalence of meeting PA guidelines, no differences were found between REACH-HF and control group at final follow-up (table 3). There was an increase in unbouted weekday MVPA and a decrease in weekday inactivity in the REACH-HF group, with the reverse found in control group, (between group differences showing a trend in favour of REACH-HF (MVPA: 15.18 (95% CI -0.32 to 30.67), p=0.06; inactivity: -21.25 (95% CI -43.24 to 0.75), p=0.06; supplementary table 2).

At post-intervention follow-up, the average change in daily MVPA (over all 7 days) in the REACH-HF group was 1.7 mins/day (minimum -76.9, maximum 177.7, SD 26.4 min/day). In the control group average MVPA change was 2.0 min/day (minimum -96.3, maximum 150.6, SD 33.1 min/day). There were no significant differences between REACH-HF group and control for all PA intensities, days (all, weekend or week), and bouted or unbouted PA, or proportion of patients meeting PA guidelines (supplementary tables 3-5).

Univariate regression analysis

Supplementary table 6 shows the associations between baseline sociodemographic, clinical and behavioural patient variables and change in MVPA at final follow-up. Older patients

For peer review only - http://bmjopen.bmj.com/site/about/guidelines.xhtml

were more likely to show a decrease in MVPA ($p<0.05$), whereas patients living with a child >18 years, with greater ISWT distance, and higher HADs anxiety score were more likely to increase their MVPA ($p<0.05$).

Post-intervention, presence of diabetes and SCHFI maintenance scores at baseline were associated with a decrease in MVPA ($p<0.05$), whereas individuals with greater ISWT distance at baseline were more likely to increase their MVPA ($p<0.05$; supplementary table 7).

Multivariable regression analysis

Table 4 shows the multivariable prediction models at final follow-up. In model 1: baseline MVPA and living with a parent was associated with a decrease in MVPA and living with a child aged >18 years was associated with an increase in MVPA. This model accounted for 15% of the variance in change in MVPA. Three patients were removed with high residual (e=211.5, e=258.3 and e=182.0) and Cook's distance d=0.35, d=0.39 and d=0.04). In model 2: baseline MVPA was associated with a decrease in MVPA, and ISWT distance and HADS anxiety score were associated with an increase in MVPA. This model accounted for 9% of the variance in MVPA change. In model 3: ISWT distance, living with a child aged over 18 years, and HADS anxiety score were strongly associated with an increase in MVPA, and living with a parent and baseline MVPA were associated with a decrease in MVPA. The model explained 15% of the variance in MVPA change at final follow-up.

Multivariable models to predict change in MVPA post-intervention are presented in supplementary table 8. In model 1: living with a parent was associated with an MVPA increase, and baseline MVPA and presence of diabetes with an MVPA decrease. This model explained 10% of the variance in MVPA change. In model 2: baseline MVPA was associated with a decrease in MVPA, and ISWT distance was most strongly associated with an increase

For peer review only - http://bmjopen.bmj.com/site/about/guidelines.xhtml

in MVPA. The model accounted for 10% of the variance in MVPA change. In model 3: living with a parent and ISWT distance were associated with MVPA increases, and baseline MVPA and presence of diabetes were strongly associated with MVPA decrease. This model accounted for 14% of the variance in MVPA change.

VIF ranged from 1.06 – 1.18 across the 6 models indicating a low level of multicollinearity.

Discussion

We undertook an in-depth analysis of RCT data of a CR intervention (REACH-HF) objectively assessing PA levels of people HF using intensity thresholds specifically developed for people with HF. When PA levels were averaged over all 7 days of the week, there was no evidence that participation in REACH-HF impacted daily PA levels compared to control. As some 48% of patients in the REACH-HF trials were already meeting UK PA guidelines at baseline (based on bouted data), it may be that there was a ceiling effect, where those already physically active would have difficulty further improving upon MVPA levels, making small changes in PA difficult to pick up statistically. This has often been the case in other CR trials.[6]

However, separating weekend and weekdays revealed important patterns in the PA response. Average weekday PA levels showed a consistent trend where MVPA and light PA increased (11 min/day and 27 min/day, respectively), and inactivity decreased (-38 min/day) in the REACH-HF group compared to control group. Over weekend days, the reverse appeared to be true, with an increase in inactivity and decrease in PA. These findings suggest that the REACH-HF participants may have compensated for increased PA during the week by being less active at the weekend, which could account for the lack of overall PA increase found.

Traditionally, CR programmes have been developed with a focus on increasing exercise capacity rather than PA promotion. REACH-HF participants were advised to exercise at least

For peer review only - http://bmjopen.bmj.com/site/about/guidelines.xhtml

3 times per week, allowing a days rest between each session, and in its current format we have shown modest positive influences of CR on PA levels of HF patients. In order to achieve greater PA benefits to patients, CR programmes may need to adapt, placing stronger emphasis on increasing unstructured daily PA behaviour. This could be facilitated by simplified PA messaging such as sit less, move more, every day, which might be easier for patients to understand and sustain in the longer term, leading to higher levels of total PA and reduced sedentary time, and is likely to have significant health benefits to HF patients.[22-24]

Given the large variation in PA differences from baseline up to final follow-up in MVPA, we were interested to investigate potential factors associated with a change in MVPA. Our results showed that particular living situations of the patient were associated with PA change (living with a parent or child >18 years), which may indicate involvement of family members is associated with PA behaviour change and self-care of HF patients.[25] Furthermore, the close relationship between exercise capacity and PA level was demonstrated in this study, where baseline MVPA and ISWT distance were the strongest and most consistent factors associated with an increase in PA levels up to 12 months follow-up, suggesting that the fitter and more active patients at baseline were more likely to increase PA levels up to 12 months follow up. To our knowledge, this is the first study to investigate the patient characteristics associated with changes in PA level and could provide early indication to potential subgroups of patients who may require more or less intensive intervention in order to maximise intervention efficacy.

Strengths and limitations

This study has a number of strengths. Firstly, the objective measurement of PA, and robust and rigorous accelerometer data processing and analysis. Literature reporting accelerometer measured PA in HF patients with CR intervention is limited, especially in large,

For peer review only - http://bmjopen.bmj.com/site/about/guidelines.xhtml

representative samples. The use of population specific intensity thresholds provides a more reliable estimate of PA levels of HF patients, compared to application of commonly used thresholds based on healthy adults.[8] However, we acknowledge that these are based on a small heterogeneous sample of patients, and is not a perfect method. Application of a single threshold for MVPA to a population will always lead to a proportion of patients PA being misclassified due to heterogeneity in exercise tolerance. Therefore, further studies are required to find an alternative approach.

This study highlights the benefit of extracting more detailed PA data, looking beyond a single PA metric (i.e. average weekly MVPA), and considering within-week differences in PA patterns. Looking at the unbouted data also provides useful information in HF patients as performing continuous bouts of exercise for 10 minutes or more may be challenging with limited exercise tolerance. However, in increasing granularity, statistical power is reduced, and future studies should consider this in sample size calculations. Future studies could also consider the distribution of PA both between- and within-days in HF patients, since research has shown that afternoon and evening PA decreases with increasing age.[25] This could inform future intervention development, targeting inactive periods throughout both the week and the day best placed for PA modification.

As this was an exploratory study, multiple repeated independent tests were conducted comparing treatment groups, and between baseline characteristics and change in MVPA. Given the dangers of multiple testing, which may have led to increased risk of Type 1 errors, our results must be treated with caution. In addition, variables associated with MVPA change were inconsistent across the two follow-up points and explained only a small proportion (10-15%) of the variance in MVPA change and combining the final follow-up data sets from 6 and 12 months may have introduced variation to the data. Whilst the sample size was sufficient for this exploratory study, some of the frequency counts of variables included in the

For peer review only - http://bmjopen.bmj.com/site/about/guidelines.xhtml

multivariable models were low. Further studies using objective PA assessment are needed to clarify the impact of exercise-based CR interventions in HF patients and the patient factors associated with change in PA.

Conclusion

This pooled analysis of randomised controlled trials shows that participation in a home-based CR intervention did not impact 7-day PA compared to control. However, participating in CR did appear to increase weekday PA levels and participants may have compensated for this by becoming more inactive during the weekend. Understanding of the behavioural effects of a home-based CR intervention could provide key targets for clinicians and researchers to adapt CR focus and PA messaging, in order to encourage patients to increase overall weekly PA and reduce inactivity. Understanding the factors associated with change in PA with CR intervention could potentially enhance development of more intense interventions specifically tailored or targeted at those with lower baseline PA or exercise capacity levels, as these patients will have the most to gain from increasing their PA.

Acknowledgments

The authors thank the patients in the REACH-HF trials and the research team for open access to the trial data. GD completed this research whilst supported by a University of Exeter Postgraduate Studentship Grant.

Authors' Contributions: GD, RT, MH and HD were involved in the study conception and interpretation of results. GD and RT led the statistical analysis. All authors provided comments on the manuscript and signed off the final version.

Funding

For peer review only

This work was supported by a University of Exeter PhD studentship with support from Professor Taylor's NIHR Senior Investigator award. Further support was received from the Medical Research Council (grant ref: MC_UU_00022/1) and the Scottish Government Chief Scientist Office (grant ref: SPHSU16).

Competing interests:

RT, HD, PD and MH were investigators in the REACH-HF study.

Data sharing statement

This study used secondary RCT data that are available through the University of Exeter's Institutional Repository, Open Research Exeter (see https://ore.exeter.ac.uk). Access to these data is permitted but controlled through requests made via the repository to the chief investigator (Professor Taylor: r.s.taylor @exeter.ac.uk). Although use is permitted, this will be on the basis that the source of the data is acknowledged (including the funder) and it includes reference to the data set 'handle'.

Ethics approval

Ethical approval was granted for the two trials by East of Scotland Research Ethics Service (15/ES/0036) and the North West Lancaster Research Ethics Committee (14/NW/1351).

References

1. Lee H, Boo S, Yu J, et al. Physical functioning, physical activity, exercise self-efficacy, and quality of life among individuals with chronic heart failure in Korea: A cross-sectional descriptive study. *J Nurs Res* 2017;25(2):131-139.

2. Hedge SM, Claggett B, Shah AM, et al. Physical activity and prognosis in the TOPCAT trial (treatments of preserved cardiac function heart failure with an aldosterone antagonist). *Circulation* 2017;136:982-992.

For peer review only - http://bmjopen.bmj.com/site/about/guidelines.xhtml

For peer review only

3. Loprinzi PD. The effects of free-living physical activity on mortality after congestive heart failure diagnosis. *Int J Cardiol* 2016;203:598-599.

4. United Kingdom Chief Medical Officer. Start active, stay active. A report on physical activity for health from the four home countries' Chief Medical Officers. 2019 https://assets.publishing.service.gov.uk/government/uploads/system/uploads/attachment_data/file/832868/uk-chief-medical-officers-physical-activity-guidelines.pdf, (Accessed 28 Feb 2022).

5. Cowie A, Buckley J, Doherty P, et al. Standards and core components for cardiovascular disease prevention and rehabilitation. *Heart* 2019;105:510-515.

6. Dibben GO, Dalal HM, Taylor RS, et al. Cardiac rehabilitation and physical activity: systematic review and meta-analysis. *Heart* 2018;104:1394-1402.

7. Alharbi M, Bauman A, Neubeck L, et al. Measuring overall physical activity for cardiac rehabilitation participants: a review of the literature. *Heart Lung Circ* 2017;26(10):1008-1025.

8. Dibben GO, Gandhi MM, Taylor RS, et al. Physical activity assessment by accelerometry in people with heart failure. *BMC Sports Sci Med Rehabil* 2020;12:47.

9. Witham MD, Argo IS, Johnston DW, et al. Predictors of exercise capacity and everyday activity in older heart failure patients. *Eur J Heart Fail* 2006;8:203-207.

10. Dibben GO, Hillsdon M, Dalal HM, et al. Factors associated with objectively assessed physical activity levels of heart failure patients. *Journal of Clinical and Experimental Cardiology* 2020;11(4):655.

For peer review only - http://bmjopen.bmj.com/site/about/guidelines.xhtml

11. Snipelisky D, Kelly J, Levine JA, et al. Accelerometer-measured daily activity in heart failure with preserved ejection fraction: clinical correlates and association with standard heart failure severity indices. *Circ Heart Fail* 2017;10:e003878.

12. Dalal HM, Zawada A, Jolly K, et al. Home based versus centre based cardiac rehabilitation: Cochrane systematic review and meta-analysis. *BMJ* 2010;340:b5631.

13. Dalal HM, Taylor RS, Greaves C, et al. Correspondence to the EJPC in response to position paper by Ambrosetti M et al. Cardiovascular rehabilitation and COVID-19: The need to maintain access to evidence-based services from the safety of home. *Eur J Prev Cardiol*; doi:10.1177/2047487320923053 Published online 30 Apr 2020.

14. Taylor RS, Hayward C, Eyre V, et al. Clinical effectiveness and cost-effectiveness of the rehabilitation enablement in chronic heart failure (REACH-HF) facilitated self-care rehabilitation intervention in heart failure patients and caregivers: rationale and protocol for a multicentre randomised controlled trial. *BMJ Open* 2015;5(12):e009994.

15. Lang CC, Smith K, Wingham J, et al. A randomised controlled trial of a facilitated home-based rehabilitation intervention in patients with heart failure with preserved ejection fraction and their caregivers: the REACH-HFpEF pilot study. *BMJ Open* 2018;8(4):e019649.

16. Eyre V, Lang CC, Smith K, et al. Rehabilitation enablement in chronic heart failure – a facilitated self-care rehabilitation intervention in patients with heart failure with preserved ejection fraction (REACH-HFpEF) and their caregivers: rationale and protocol for a single-centre pilot randomised controlled trial. *BMJ Open* 2016;6(10):e012853.

17. Dalal HM, Taylor RS, Jolly K, et al. The effects and costs of home-based rehabilitation for heart failure with reduced ejection fraction: The REACH-HF multicentre randomized controlled trial. *Eur J Prev Cardiol* 2019;26(3):262-272.

For peer review only - http://bmjopen.bmj.com/site/about/guidelines.xhtml

For peer review only

18. van Hees VT, Gorzelniak L, León EC, et al. Separating movement and gravity components in an acceleration signal and implications for the assessment of human daily physical activity. *PLOS One* 2013;8(4):e61691.

19. van Hees VT, Fang Z, Langford J, et al. Autocalibration of accelerometer data for free-living physical activity assessment using local gravity and temperature: an evaluation on four continents. *J Appl Physiol* 1985;117:738-744.

20. Bakrania K, Yates T, Rowlands AV, et al. Intensity thresholds on raw acceleration data: Euclidean norm minus one (ENMO) and mean amplitude deviation (MAD) approaches. *PLoS ONE* 2016;11(10):e0164045.

21. Sabia S, van Hees VT, Shipley MJ, et al. Association between questionnaire- and accelerometer assessed physical activity: the role of sociodemographic factors. *Am J Epidemiol* 2014; 179:781-790.

22. Ekelund U, Tarp J, Steene-Johannessen J, et al. Dose-response associations between accelerometry measured physical activity and sedentary time and all cause mortality: systematic review and harmonised meta-analysis. *BMJ* 2019;366:l4570.

23. Buijs DM, Ramadi A, MacDonald K, et al. Quantity and quality of daily physical activity in older cardiac patients. *Canadian Journal of Cardiovascular Nursing* 2015;25(3):10-16.

24. Schrack JA, Zipunnikov V, Goldsmith J, et al. Assessing the “physical cliff”: detailed quantification of age-related differences in daily patterns of physical activity. *J Gerontol A Biol Sci Med Sci* 2014;69(8):973-979.

25. Wingham J, Harding G, Britten N, Dalal H. Heart failure patients’ attitudes, beliefs, expectations and experiences of self-management strategies: a qualitative synthesis. *Chronic Illn* 2013;10(2):135–54.

For peer review only - http://bmjopen.bmj.com/site/about/guidelines.xhtml

Legends

Figure 1: participant flow diagram

For peer review only

For peer review only - http://bmjopen.bmj.com/site/about/guidelines.xhtml

Appendices

Supplementary materials provided in a separate document

Tables

For peer review only

For peer review only - http://bmjopen.bmj.com/site/about/guidelines.xhtml

Table 1: Summary baseline patient characteristics. Data are presented as N(%) unless otherwise stated.

	REACH-HF (N=122)	Control (N=125)
Mean (SD) Age (years)	70.5 (10.0)	71.3 (10.7)
Female Sex	40 (33)	30 (24)
Mean (SD) BMI (kg/m^2)	29.9 (6.6)	30.0 (5.9)
Ethnicity (white vs other)	7 (6)	4 (3)
Type of HF (HFrEF vs HFpEF)	24 (20)	25 (20)
NYHA class		
NYHA I	24 (20)	16 (13)
NYHA II	71 (58)	76 (61)
NYHA III-IV	27 (22)	33 (26)
Mean (SD) LVEF (%)	38.4 (14.7)	38.1 (15.5)
Mean (SD) NT-proBNP (pg/ml)	1288.3 (1794.3)	1364.4 (1602.1)
Trial site		
Truro	27 (22)	29 (23)
Gwent	22 (18)	22 (18)
Birmingham	24 (20)	24 (19)
York	25 (20)	25 (20)
Dundee	24 (20)	25 (20)
Total number of comorbidities* (median, range)	3 (2-5)	3 (2-5)
Mean (SD) ISWT (peak distance, m)	241.9 (157.1)	219.1 (144.2)
Mean (SD) MLHFQ		

For peer review only - http://bmjopen.bmj.com/site/about/guidelines.xhtml

Overall	33.5 (24.5)	30.7 (23.0)
Physical	17.3 (11.8)	16.1 (11.6)
Emotional	7.7 (7.7)	7.1 (7.0)
Mean (SD) HADS		
Anxiety	5.3 (4.5)	5.9 (4.4)
Depression	4.8 (3.6)	4.8 (3.4)
Mean (SD) HeartQoL		
Global	1.8 (0.8)	1.8 (0.8)
Physical	1.6 (0.8)	1.6 (0.8)
Emotional	2.1 (0.9)	2.1 (0.9)
Mean (SD) EQ-5D-5L	0.7 (0.3)	0.7 (0.3)
Mean (SD) SCHFI		
Maintenance	56.0 (16.1)	53.0 (15.4)
Management	42.0 (24.7)	39.6 (20.4)
Confidence	61.9 (25.2)	63.6 (23.4)

REACH-HF: Rehabilitation enablement in chronic heart failure; SD: standard deviation; BMI: body mass index; NYHA: New York Heart Association; HF: heart failure; LVEF: left ventricular ejection fraction; NT-proBNP: N-terminal proB-type natriuretic peptide; HFrEF: heart failure with reduced ejection fraction; HFpEF: heart failure with preserved ejection fraction; ISWT: incremental shuttle walk test; MLHFQ: Minnesota living with heart failure questionnaire; HADS: hospital anxiety and depression scale; SCHFI: self-care in heart failure index.

* Comorbidities including: angina, diabetes, myocardial infarction, hypertension, osteoporosis, stroke, asthma, chronic back pain, chronic renal impairment, arthritis, atrial fibrillation, chronic obstructive pulmonary disease, depression.

For peer review only

Table 2: Intervention effects on PA outcomes at final follow-up

	Baseline		Final follow-up		Δ to final follow-up		Between group difference (mean, 95% CI) p-value
	REACH-HF mean (SD, N)	Control mean (SD, N)	REACH-HF mean (SD, N)	Control mean (SD, N)	REACH-HF mean (SD, N)	Control mean (SD, N)	
All days Bouted							
MVPA (min/day)	43.62 (51.15, 80)	49.66 (73.98, 93)	47.61 (67.78, 80)	44.56 (72.10, 93)	3.99 (51.53, 80)	-5.10 (34.23, 93)	7.61 (-5.11 to 20.33) p=0.24
Light (min/day)	213.80 (110.94, 80)	219.93 (114.73, 93)	221.92 (129.57, 80)	209.50 (120.90, 93)	8.12 (87.01, 80)	-10.43 (87.85, 93)	16.87 (-8.79 to 42.54) p=0.20
Inactive (min/day)	1182.58 (144.43, 80)	1170.41 (164.42, 93)	1170.47 (170.47, 80)	1185.78 (167.52, 93)	-12.12 (115.83, 80)	15.53 (10.52, 93)	-24.77 (-56.69 to 7.16) p=0.13
Weekend Days Bouted							
MVPA (min/day)	43.72 (56.62, 80)	41.0 (69.84, 93)	38.67 (61.04, 80)	36.43 (62.10, 93)	-5.05 (52.27, 80)	-4.57 (43.31, 93)	0.14 (-12.92 to 13.21) p=0.98
Light (min/day)	208.10 (123.23, 80)	202.18 (118.25, 93)	201.36 (128.36, 80)	206.22 (123.41, 93)	-6.75 (107.42, 80)	4.04 (103.72, 93)	-8.38 (-37.96 to 21.20) p=0.58

For peer review only - http://bmjopen.bmj.com/site/about/guidelines.xhtml

For peer review only

Inactive (min/day)	1188.18 (161.84, 80)	1196.82 (160.52, 93)	1199.98 (162.57, 80)	1197.35 (158.21, 93)	11.80 (131.33, 80)	0.53 (121.78, 93)	8.27 (-27.01 to 43.55) p=0.64
Week Days Bouted							
MVPA (min/day)	43.58 (50.71, 80)	53.12 (79.66, 93)	51.19 (71.70, 80)	47.82 (78.90, 93)	7.61 (55.14, 80)	-5.31 (37.46, 93)	10.87 (-2.94 to 24.69) p=0.12
Light (min/day)	216.08 (112.68, 80)	227.03 (120.85, 93)	230.15 (135.37, 80)	210.81 (125.37, 93)	14.07 (90.71, 80)	-16.22 (92.74, 93)*	26.87 (-0.05 to 53.78) p=0.05
Inactive (min/day)	1180.34 (143.59, 80)	1159.85 (174.23, 93)	1158.66 (178.56, 80)	1181.37 (178.59, 93)	-21.68 (122.78, 80)	21.53 (106.40, 93)*	-38.31 (-72.13 to -4.50) p=0.03

REACH-HF: Rehabilitation enablement in chronic heart failure; SD: standard deviation; MVPA: moderate-to-vigorous physical activity; PA: physical activity

* p<0.05 REACH-HF group vs control

For peer review only - http://bmjopen.bmj.com/site/about/guidelines.xhtml

Table 3: Intervention effects on proportion of patients meeting PA guidelines at final follow-up

	Baseline		Final follow-up		OR (95% CI) p-value
	REACH-HF (n, N, %)	Control (n, N, %)	REACH-HF (n, N, %)	Control (n, N, %)	
Bouted					
Proportion meeting guidelines	42, 80, 53%	45, 93, 48%	43, 80, 54%	39, 93, 42%	0.43 (0.16 to 1.14) p=0.09
Unbouted					
Proportion meeting guidelines	80, 80, 100%	93, 93, 100%	79, 80, 99%	93, 93, 100%	-

REACH-HF: Rehabilitation enablement in chronic heart failure; OR: odds ratio

For peer review only - http://bmjopen.bmj.com/site/about/guidelines.xhtml

Table 4: Comparison of multivariable models to predict change in minutes/day MVPA at final follow-up.

Multivariable model	Variables included in model (p<0.05)	Unstandardized beta coefficient (95% CI)	t-statistic	Variable P-value	Model Adjusted R^2 (p-value)
1. Socio-demographic	Group	-4.03 (-12.19 to 4.12)	-0.98	0.33	0.15 (<0.001)
	Baseline MVPA	-0.15 (-0.22 to -0.09)	-4.64	<0.001	
	Centre	-0.28 (-3.0 to 2.45)	-0.20	0.84	
	BNP 2000	-6.58 (-17.27 to 4.10)	-1.22	0.23	
	Live with parent	-41.81 (-72.96 to -10.67)	-2.65	0.009	
	Live with child over 18	16.04 (0.05 to 32.02)	1.98	0.049	
	constant	5.89 (-4.92 to 16.70)	1.08	0.28	
2. Exercise capacity and health status	Group	-6.72 (-19.81 to 6.37)	-1.01	0.31	0.09 (0.001)
	Baseline MVPA	-0.20 (-0.31 to -0.09)	-3.47	0.001	
	Centre	0.13 (-4.34 to 4.60)	0.06	0.95	
	BNP 2000	-7.39 (-25.08 to 10.30)	-0.83	0.41	
	ISWT peak	0.07 (0.02 to 0.11)	2.69	0.008	

For peer review only - http://bmjopen.bmj.com/site/about/guidelines.xhtml

	HADs anxiety	1.90 (0.41 to 3.38)	2.52	0.013	
	constant	-12.91 (-37.03 to 11.21)	-1.06	0.29	
3. Socio-demographic, exercise capacity and health status*	Group	-8.37 (-21.13 to 4.38)	-1.3	0.20	0.15 (<0.001)
	Baseline MVPA	-0.21 (-0.33 to -0.10)	-3.73	<0.001	
	Centre	-0.11 (-4.47 to 4.25)	-0.05	0.96	
	BNP 2000	-6.21 (-23.44 to 11.01)	-0.71	0.48	
	ISWT peak	0.08 (0.03 to 0.12)	3.16	0.002	
	Live with child >18	30.48 (5.92 to 55.04)	2.45	0.02	
	HADS anxiety	1.89 (0.44 to 3.33)	2.58	0.01	
	Live with parent	-52.60 (-100.16 to -5.05)	-2.19	0.03	
	constant	-14.51 (-38.05 to 9.04)	-1.22	0.23	

MVPA: moderate-to-vigorous physical activity; BNP 2000: NT-proBNP above or below 2000 pg/ml; ISWT: incremental shuttle walk test; HADS: hospital anxiety and depression score

* all variables p<0.05 from multivariate models 1 and 2

For peer review only - http://bmjopen.bmj.com/site/about/guidelines.xhtml

Figures

Provided separately.

For peer review only

For peer review only - http://bmjopen.bmj.com/site/about/guidelines.xhtml

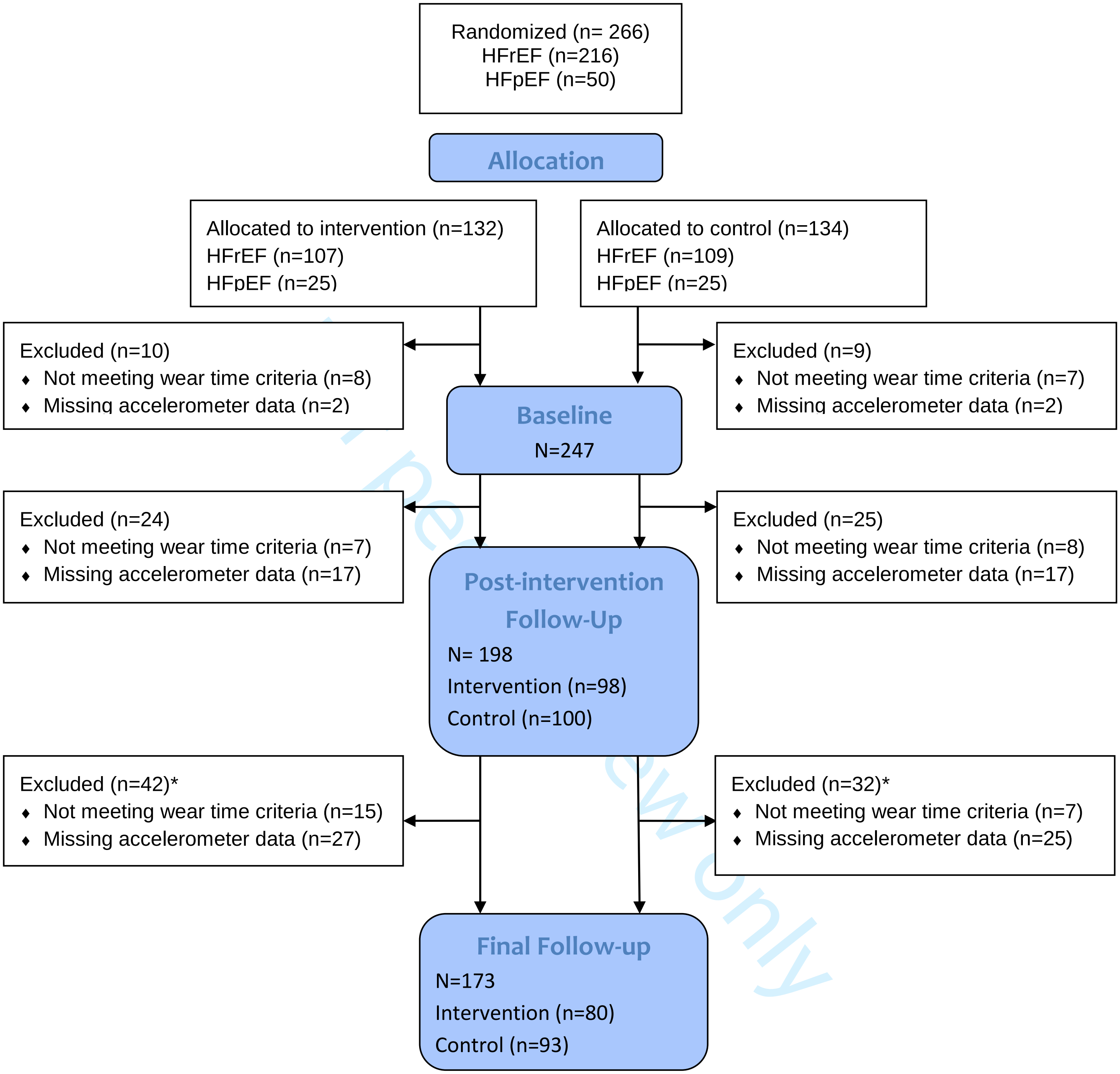

*14 patients excluded from post-intervention follow-up analysis (for missing data or not enough valid days) were included in the final follow-up with sufficient accelerometer data.

Supplementary tables

Supplementary table 1: Patient characteristics at baseline. Data are presented as N(%) unless otherwise stated.

	REACH-HF N=122	Control N=125
Mean (SD) Age (years)	70.5 (10.0)	71.3 (10.7)
Female Sex	40 (33)	30 (24)
Mean (SD) BMI (kg/m^2)	29.9 (6.6)	30.0 (5.9)
Employment status		
In employment/ Self-employed	15 (12)	16 (13)
Retired	98 (80)	101 (81)
Housework	0 (0)	1 (1)
Unemployed	7 (6)	3 (2)
Other	2 (2)	4 (3)
Ethnicity (white vs other)	7 (6)	4 (3)
NYHA class		
NYHA I	24 (20)	16 (13)
NYHA II	71 (58)	76 (61)
NYHA III-IV	27 (22)	33 (26)
Time since HF diagnosis		
0 years	37 (30)	37 (30)
1 year	24 (20)	23 (18)
2 years	61 (50)	65 (52)
Cause of HF		
Ischaemic	52 (43)	63 (50)
Non-ischaemic	63 (52)	53 (43)
Not known/ classified	7 (5)	9 (7)
Mean (SD) LVEF (%)	38.4 (14.7)	38.1 (15.5)
Mean (SD) NT-proBNP (pg/ml)	1288.3 (1794.3)	1364.4 (1602.1)
Living alone	32 (26)	34 (27)
Living with partner	79 (65)	79 (63)
Living with child>18	7 (6)	10 (8)
Living with child<18	3 (2)	2 (2)
Living with parent	2 (2)	3 (2)
Smoking history		
Current smoker	7 (6)	7 (5)
Ex-smoker	67 (55)	72 (58)
Never smoked	48 (39)	46 (37)
Trial site		
Truro	27 (22)	29 (23)
Gwent	22 (18)	22 (18)
Birmingham	24 (20)	24 (19)
York	25 (20)	25 (20)
Dundee	24 (20)	25 (20)
Comorbidities		
Angina	32 (26)	33 (26)
Diabetes	31 (25)	28 (22)
MI	29 (24)	41 (33)
Hypertension	59 (48)	54 (43)
Osteoporosis	11 (9)	6 (5)

For peer review only

Stroke	14 (11)	15 (12)
Asthma	14 (11)	14 (11)
Chronic back pain	40 (33)	36 (29)
Chronic renal impairment	17 (14)	28 (22)
Arthritis	57 (47)	50 (40)
Atrial fibrillation	51 (42)	67 (54)
COPD	13 (11)	14 (11)
Depression	30 (25)	31 (25)
Total number of comorbidities	3 (2-5)	3 (2-5)
Medication		
Angiotensin II receptor antagonist	38 (31)	27 (22)
ACE inhibitor	71 (58)	84 (67)
Aldosterone antagonist	62 (51)	56 (45)
Anticoagulant	52 (43)	62 (50)
Beta blocker	99 (81)	95 (76)
Digoxin	23 (19)	16 (13)
Ivabradine	5 (4)	8 (6)
Loop diuretic	83 (68)	87 (70)
Nitrate	22 (18)	16 (13)
Thiazide diuretic	1 (1)	3 (2)
Type of HF (HFrEF vs HFpEF)	24 (20)	25 (20)
Mean (SD) ISWT (peak distance, m)	241.9 (157.1)	219.1 (144.2)
Mean (SD) MLHFQ		
Overall	33.5 (24.5)	30.7 (23.0)
Physical	17.3 (11.8)	16.1 (11.6)
Emotional	7.7 (7.7)	7.1 (7.0)
Mean (SD) HADS		
Anxiety	5.3 (4.5)	5.9 (4.4)
Depression	4.8 (3.6)	4.8 (3.4)
Mean (SD) HeartQoL		
Global	1.8 (0.8)	1.8 (0.8)
Physical	1.6 (0.8)	1.6 (0.8)
Emotional	2.1 (0.9)	2.1 (0.9)
Mean (SD) EQ-5D-5L	0.7 (0.3)	0.7 (0.3)
Mean (SD) SCHFI		
Maintenance	56.0 (16.1)	53.0 (15.4)
Management	42.0 (24.7)	39.6 (20.4)
Confidence	61.9 (25.2)	63.6 (23.4)

REACH-HF: Rehabilitation enablement in chronic heart failure; SD: standard deviation; BMI: body mass index; NYHA: New York Heart Association; HF: heart failure; LVEF: left ventricular ejection fraction; NT-proBNP: N-terminal proB-type natriuretic peptide; MI: myocardial infection; COPD: chronic obstructive pulmonary disease; HFrEF: heart failure with reduced ejection fraction; HFpEF: heart failure with preserved ejection fraction; ISWT: incremental shuttle walk test; MLHFQ: Minnesota living with heart failure questionnaire; HADS: hospital anxiety and depression scale; SCHFI: self-care in heart failure index.

For peer review only

Supplementary table 2: Intervention effects at final follow-up, unbouted PA data

	Baseline		Final follow-up		Δ to final follow-up		Between group difference (mean, 95% CI) p-value
	REACH-HF mean (sd, N)	Control mean (sd, N)	REACH-HF mean (sd, N)	Control mean (sd, N)	REACH-HF mean (sd, N)	Control mean (sd, N)	
All days unbouted							
MVPA (min/day)	185.98 (86.30, 80)	190.47 (96.18, 93)	186.76 (97.24, 80)	180.30 (97.22, 93)	0.78 (55.62, 80)	-10.17 (43.89, 93)	9.86 (-4.96 to 24.69) p=0.19
Light (min/day)	195.39 (48.24, 80)	194.29 (46.69, 93)	193.36 (52.48, 80)	189.97 (46.66, 93)	-2.03 (33.65, 80)	-4.32 (34.66, 93)	2.35 (-7.65 to 12.35) p=0.64
Inactive (min/day)	1058.63 (108.27, 80)	1055.24 (118.54, 93)	1059.88 (121.89, 80)	1069.73 (122.72, 93)	1.25 (73.95, 80)	14.49 (68.52, 93)	-11.97 (-33.12 to 9.18) p=0.27
Weekend days unbouted							
MVPA (min/day)	183.15 (91.01, 80)	175.14 (91.13, 93)	173.76 (94.23, 80)	169.86 (91.67, 93)	-9.39 (62.73, 80)	-5.28 (54.92, 93)	-2.87 (-19.97 to 14.23) p=0.74
Light (min/day)	193.14 (54.28, 80)	188.28 (49.54, 93)	185.07 (55.34, 80)	189.01 (50.88, 93)	-8.07 (47.07, 80)	0.73 (45.62, 93)	-6.97 (-19.89 to 5.94) p=0.29
Inactive (min/day)	1063.71 (177.88, 80)	1076.58 (120.30, 98)	1081.17 (125.01, 80)	1081.13 (118.25, 93)	17.46 (92.05, 80)	4.55 (83.95, 93)	10.07 (-15.22 to 35.35) p=0.43
Week days unbouted							
MVPA (min/day)	187.11 (86.72, 80)	196.60 (101.66, 93)	191.96 (100.34, 80)	184.47 (102.63, 93)	4.85 (58.35, 80)	-12.13 (45.46, 93)*	15.18 (-0.32 to 30.67) p=0.06
Light (min/day)	196.29 (48.94, 80)	196.70 (49.06, 93)	196.68 (53.23, 80)	190.36 (48.31, 93)	0.39 (33.02, 80)	-6.34 (37.88, 93)	6.30 (-4.05 to 16.65) p=0.23
Inactive (min/day)	1056.60 (109.35, 80)	1046.70 (123.47, 93)	1051.36 (124.37, 80)	1065.17 (129.8, 93)	-5.24 (76.14, 80)	18.47 (72.05, 93)*	-21.25 (-43.24 to 0.75) p=0.06

REACH-HF: Rehabilitation enablement in chronic heart failure; SD: standard deviation; MVPA: moderate-to-vigorous physical activity; PA: physical activity
* p<0.05 REACH-HF group vs control

For peer review only

Supplementary table 3: Intervention effects on PA outcomes at post-intervention follow-up, bouted PA data

	Baseline		Post-intervention		Δ to post-intervention		Between group difference (mean, 95% CI) p-value
	REACH-HF mean (sd, N)	Control mean (sd, N)	REACH-HF mean (sd, N)	Control mean (sd, N)	REACH-HF mean (sd, N)	Control mean (sd, N)	
All days bouted							
MVPA (min/day)	37.90 (47.51, 98)	44.96 (65.58, 100)	39.64 (51.15, 98)	46.96 (72.11, 100)	1.74 (26.42, 98)	2.00 (33.08, 100)	-0.76 (-9.26 to 7.74) p=0.86
Light (min/day)	197.91 (103.22, 98)	219.30 (108.05, 100)	198.23 (105.26, 98)	213.05 (114.25, 100)	0.31 (70.48, 98)	-6.25 (76.81, 100)	0.98 (-19.09 to 21.06) p=0.92
Inactive (min/day)	1204.19 (133.00, 98)	1175.74 (151.00, 100)	1202.14 (140.26, 98)	1179.99 (164.00, 100)	-2.05 (76.55, 98)	4.25 (92.03, 100)	-2.40 (-26.20 to 21.40) p=0.84
Weekend days bouted							
MVPA (min/day)	39.01 (53.54, 98)	37.03 (56.49, 100)	36.62 (53.77, 98)	43.99 (66.40, 100)	-2.39 (33.09, 98)	6.96 (50.56, 100)	-9.12 (-20.82 to 2.57) p=0.13
Light (min/day)	194.02 (116.16, 98)	198.50 (111.91, 100)	182.82 (109.02, 98)	192.50 (113.65, 100)	-11.21 (88.87, 98)	-6.00 (102.58, 100)	-7.63 (-31.90 to 16.64) p=0.54
Inactive (min/day)	1206.97 (148.83, 98)	1204.48 (145.62, 100)	1220.56 (144.51, 98)	1203.51 (155.21, 100)	13.59 (96.46, 98)	-0.97 (126.04, 100)	15.84 (-13.92 to 45.59) p=0.30
Week days bouted							
MVPA (min/day)	37.46 (47.03, 98)	48.13 (72.86, 100)	40.85 (53.13, 98)	48.15 (77.36, 100)	3.39 (30.04, 98)	0.01 (36.18, 100)	2.26 (-7.16 to 11.67) p=0.64
Light (min/day)	199.47 (104.88, 98)	227.63 (114.60, 100)	204.39 (108.06, 98)	221.27 (120.74, 100)	4.92 (75.09, 98)	-6.35 (81.07, 100)	3.39 (-17.91 to 24.70) p=0.75
Inactive (min/day)	1203.07 (133.15, 98)	1164.24 (161.83, 100)	1194.76 (143.28, 98)	1170.58 (174.37, 100)	-8.31 (85.00, 98)	6.34 (96.79, 100)	-13.53 (-39.31 to 12.25) p=0.30)

REACH-HF: Rehabilitation enablement in chronic heart failure; SD: standard deviation; MVPA: moderate-to-vigorous physical activity; PA: physical activity

For peer review only

Supplementary table 4: Intervention effects at post-intervention follow-up, unbouted PA data

	Baseline		Post intervention		Δ to post-intervention		Between group difference (mean, 95% CI) p-value
	REACH-HF mean (sd, N)	Control mean (sd, N)	REACH-HF mean (sd, N)	Control mean (sd, N)	REACH-HF mean (sd, N)	Control mean (sd, N)	
All days Unbouted							
MVPA (min/day)	171.51 (79.28, 98)	185.77 (88.79, 100)	170.34 (83.87, 98)	182.84 (95.19, 100)	-1.17 (37.95, 98)	-2.93 (44.70, 100)	0.58 (-11.17 to 12.34) p=0.92
Light (min/day)	191.83 (47.69, 98)	194.95 (46.02, 100)	189.74 (46.55, 98)	190.66 (41.19, 100)	-2.09 (32.45, 98)	-4.28 (32.49, 100)	1.14 (-7.18 to 9.46) p=0.79
Inactive (min/day)	1076.66 (102.53, 98)	1059.28 (110.43, 100)	1079.92 (106.13, 98)	1066.50 (114.70, 100)	3.27 (60.16, 98)	7.22 (68.83, 100)	-0.65 (-18.56 to 17.26) p=0.94
Weekend days Unbouted							
MVPA (min/day)	169.77 (84.81, 98)	170.92 (82.42, 100)	160.49 (86.13, 98)	171.80 (92.20, 100)	-9.28 (45.26, 98)	0.88 (63.94, 100)	-10.48 (-25.78 to 4.81) p=0.18
Light (min/day)	189.50 (53.00, 98)	187.11 (49.14, 100)	181.25 (52.40, 98)	181.35 (42.84, 100)	-8.25 (46.60, 98)	-5.76 (42.95, 100)	-1.75 (-12.71 to 9.20) p=0.75
Inactive (min/day)	1080.73 (113.23, 98)	1081.98 (111.68, 100)	1098.26 (115.25, 98)	1086.85 (114.35, 100)	17.53 (79.97, 98)	4.88 (93.05, 100)	13.10 (-9.90 to 36.11) p=0.26
Week days Unbouted							
MVPA (min/day)	172.20 (79.63, 98)	191.72 (94.89, 100)	174.28 (85.32, 98)	187.26 (99.35, 100)	2.07 (42.27, 98)	-4.46 (46.60, 100)	4.29 (-8.27 to 16.86) p=0.50
Light (min/day)	192.77 (48.69, 98)	198.08 (48.26, 100)	193.14 (46.97, 98)	194.39 (43.64, 100)	0.37 (33.14, 98)	-3.69 (34.36, 100)	2.34 (-6.30 to 10.98) p=0.59
Inactive (min/day)	1075.03 (103.46, 98)	1050.20 (115.91, 100)	1072.59 (106.80, 98)	1058.36 (119.56, 100)	-2.44 (64.34, 98)	8.15 (71.34, 100)	-5.43 (-24.22 to 13.36) p=0.57

REACH-HF: Rehabilitation enablement in chronic heart failure; SD: standard deviation; MVPA: moderate-to-vigorous physical activity; PA: physical activity

For peer review only

Supplementary table 5: Intervention effects on proportion of patients meeting PA guidelines at post-intervention follow-up

	Baseline		Post-intervention		OR (95% CI) p-value
	REACH-HF (n, N, %)	Control (n, N, %)	REACH-HF (n, N, %)	Control (n, N, %)	
Bouted Proportion meeting guidelines	47, 98, 48%	47, 100, 47%	49, 98, 50%	47, 100, 47%	0.79 (0.34 to 1.84) p=0.59
Unbouted Proportion meeting guidelines	98, 98, 100%	100, 100, 100%	96, 98, 98%	100, 100, 100%	-

REACH-HF: Rehabilitation enablement in chronic heart failure; OR: odds ratio

For peer review only

Supplementary table 6: Univariate association with change in MVPA at final follow-up, controlling for trial stratifiers, group and baseline MVPA

Sociodemographic, exercise capacity and health status variables<br>N=173 unless otherwise stated	Unstandardized beta coefficient (95% CI)	p-value
Age	-0.77 (-1.49 to -0.05)	0.04
Gender	1.19 (-13.50 to 15.89)	0.87
BMI	-0.46 (-1.68 to 0.75)	0.45
Employment status		
In employment/Self-employed	Comparison group	0.26
Retired	-17.13 (-38.46 to 4.19)	
Housework	-18.44 (-105.09 to 68.21)	
Unemployed	-39.19 (-82.28 to 4.41)	
Other	-38.71 (-84.28 to 6.86)	
Ethnicity (white vs other)	-20.33 (-56.51 to 15.86)	0.27
NYHA class		
NYHA I	Comparison group	0.10
NYHA II	-17.20 (-34.54 to 0.14)	
NYHA III-IV	-21.84 (-43.34 to -0.34)	
Time since HF diagnosis		
0 years	Comparison group	0.27
1 year	-11.12 (-30.01 to 7.76)	
2 years	-11.72 (-26.48 to 3.03)	
Cause of HF		
Ischaemic	Comparison group	0.44
Non-ischaemic	8.53 (-4.84 to 21.90)	
Not known/classified	0.91 (-30.46 to 32.29)	
LVEF (%) (N=137)	-0.52 (-1.57 to 0.52)	0.33
NT-proBNP (pg/ml)	-0.003 (-0.009 to 0.004)	0.42
Living alone	-3.06 (-18.40 to 12.28)	0.69
Living with partner	-3.06 (-18.40 to 12.28)	0.69
Living with child >18	25.98 (1.80 to 50.16)	0.04
Living with child <18	-1.16 (-50.64 to 48.33)	0.96
Living with parent	-45.62 (-94.86 to 3.62)	0.07
Smoking history		
Current smoker	Comparison group	0.61
Ex-smoker	16.14 (-16.57 to 48.84)	
Never smoked	16.28 (-17.23 to 49.80)	
Trial site		
Truro	Comparison group	0.17
Gwent	-9.09 (-27.90 to 9.72)	
Birmingham	15.20 (-5.94 to 36.35)	
York	-2.93 (-21.72 to 15.87)	
Dundee	-10.67 (-29.41 to 8.07)	

For peer review only

Comorbidities		
Angina	0.16 (-15.50 to 15.82)	0.98
Diabetes	-12.85 (-27.80 to 2.09)	0.09
MI	-5.75 (-20.12 to 8.62)	0.43
Hypertension	-1.92 (-15.17 to 11.33)	0.78
Osteoporosis	6.64 (-19.25 to 32.52)	0.61
Stroke	-7.21 (-27.07 to 12.64)	0.47
Asthma	4.16 (-18.08 to 26.41)	0.71
Chronic back pain	1.60 (-11.91 to 15.11)	0.82
Chronic renal impairment	-3.58 (-21.05 to 13.89)	0.69
Arthritis	9.77 (-3.38 to 22.91)	0.14
Atrial fibrillation	-10.54 (-23.55 to 2.47)	0.11
COPD	-15.62 (-35.03 to 3.79)	0.11
Depression	-2.0 (-17.05 to 13.06)	0.79
Total number of comorbidities	-1.29 (-4.45 to 1.87)	0.42
Total number of cardiorespiratory and metabolic comorbidities*	-4.56 (-9.47 to 0.35)	0.07
Total number of physical and musculoskeletal comorbidities†	3.85 (-3.25 to 10.95)	0.29
Medication		
Angiotensin II receptor antagonist	4.66 (-9.91 to 19.23)	0.53
ACE inhibitor	-0.59 (-14.16 to 12.98)	0.93
Aldosterone antagonist	4.13 (-9.52 to 17.78)	0.55
Anticoagulant	-7.62 (-20.70 to 5.47)	0.25
Beta blocker	-7.79 (-24.33 to 8.75)	0.35
Digoxin	-0.52 (-18.20 to 17.16)	0.95
Ivabradine	5.48 (-25.45 to 36.42)	0.73
Loop diuretic	-12.65 (-26.68 to 1.39)	0.08
Nitrate	-1.50 (-21.28 to 18.27)	0.88
Thiazide diuretic	-15.84 (-65.32 to 33.65)	0.53
Type of HF (HFrEF vs HFpEF)	collinearity	
ISWT (peak distance) (N=165) baseline	0.06 (0.01 to 0.12)	0.02
Overall ENMO	1.44 (-0.52 to 3.39)	0.15
MLHFQ	0.04 (-0.26 to 0.33)	0.81
Overall		
Physical	-0.14 (-0.74 to 0.47)	0.66
Emotional	0.47 (-0.43 to 1.36)	0.31
HADS		
Anxiety	1.83 (0.40 to 3.25)	0.01
Depression	-0.13 (-2.27 to 2.01)	0.91
HeartQoL		
Global	-2.09 (-11.01 to 6.82)	0.64
Physical	-0.66 (-9.02 to 7.69)	0.88
Emotional	-3.82 (-11.27 to 3.64)	0.31
EQ-5D-5L (N=172)	4.58 (-22.35 to 31.52)	0.74

For peer review only

SCHFI		
Maintenance	0.19 (-0.25 to 0.64)	0.39
Management (N=94)	-0.01 (-0.28 to 0.25)	0.92
Confidence	0.01 (-0.26 to 0.27)	0.96

BMI: body mass index; NYHA: New York Heart Association; HF: heart failure; LVEF: left ventricular ejection fraction; NT-proBNP: N-terminal proB-type natriuretic peptide; MI: myocardial infection; COPD: chronic obstructive pulmonary disease; HFrEF: heart failure with reduced ejection fraction; HFpEF: heart failure with preserved ejection fraction; ISWT: incremental shuttle walk test; ENMO: Euclidean norm minus one; MLHFQ: Minnesota living with heart failure questionnaire; HADS: hospital anxiety and depression scale; SCHFI: self-care in heart failure index.

For peer review only

Supplementary table 7: Univariate association with change in MVPA at post-intervention follow-up, controlling for trial stratifiers, group and baseline MVPA

Sociodemographic, exercise capacity and health status variables N=198 unless otherwise stated	Unstandardized beta coefficient (95% CI)	p-value
Age	-0.39 (-0.85 to 0.08)	0.11
Gender	-5.74 (-15.85 to 4.37)	0.26
BMI	-0.19 (-1.00 to 0.63)	0.65
Employment status		
In employment/Self-employed	Comparison group	0.30
Retired	-10.83 (-25.08 to 3.41)	
Housework	-10.65 (-72.68 to 51.38)	
Unemployed	-29.16 (-56.92 to -1.40)	
Other	-3.58 (-32.96 to 25.81)	
Ethnicity (white vs other)	-1.91 (-27.60 to 23.77)	0.88
NYHA class		
NYHA I	Comparison group	0.17
NYHA II	-5.67 (-17.94 to 6.59)	
NYHA III-IV	-13.58 (-28.25 to 1.08)	
Time since HF diagnosis		
0 years	Comparison group	0.80
1 year	-3.15 (-16.06 to 9.76)	
2 years	-3.30 (-13.30 to 6.70)	
Cause of HF		
Ischaemic	Comparison group	0.78
Non-ischaemic	-2.90 (-11.93 to 6.12)	
Not known/classified	1.62 (-18.58 to 21.82)	
LVEF (%) (N=151)	-0.08 (-0.67 to 0.51)	0.79
NT-proBNP (pg/ml)	-0.0003 (-0.004 to 0.003)	0.87
Living alone	-5.25 (-15.76 to 5.25)	0.33
Living with partner	2.94 (-6.55 to 12.42)	0.61
Living with child >18	2.94 (-14.88 to 20.76)	0.33
Living with child <18	5.81 (-29.66 to 41.29)	0.32
Living with parent	25.68 (-1.81 to 53.16)	0.07
Smoking history		
Current smoker	Comparison group	0.21
Ex-smoker	6.47 (-14.09 to 27.04)	
Never smoked	-1.55 (-22.93 to 19.83)	
Trial site		
Truro	Comparison group	0.76
Gwent	4.19 (-8.66 to 17.03)	
Birmingham	5.20 (-8.71 to 19.12)	
York	-1.81 (-14.46 to 10.84)	
Dundee	-2.57 (-15.59 to 10.46)	

For peer review only

Comorbidities		
Angina	-1.72 (-11.93 to 8.48)	0.74
Diabetes	-12.67 (-22.52 to 2.81)	0.01
MI	3.43 (-6.40 to 12.26)	0.49
Hypertension	-2.67 (-11.57 to 6.23)	0.55
Osteoporosis	-10.64 (-29.90 to 8.61)	0.28
Stroke	-1.65 (-15.44 to 12.14)	0.81
Asthma	8.83 (-5.38 to 23.04)	0.22
Chronic back pain	-8.46 (-17.87 to 0.94)	0.08
Chronic renal impairment	0.01 (-0.05 to 0.07)	0.68
Arthritis	-2.62 (-11.67 to 6.43)	0.57
Atrial fibrillation	-2.47 (-11.36 to 6.42)	0.58
COPD	-4.18 (-18.58 to 10.21)	0.57
Depression	-3.33 (-13.99 to 7.32)	0.54
Total number of comorbidities	-1.05 (-3.13 to 1.04)	0.32
Total number of cardiorespiratory and metabolic comorbidities*	-1.77 (-5.04 to 1.51)	0.29
Total number of physical and musculoskeletal comorbidities†	-3.33 (-8.37 to 1.71)	0.19
Medication		
Angiotensin II receptor antagonist	-0.99 (-10.74 to 8.76)	0.84
ACE inhibitor	5.54 (-3.47 to 14.56)	0.23
Aldosterone antagonist	3.87 (-5.29 to 13. 03)	0.83
Anticoagulant	-8.31 (-17.13 to 0.51)	0.07
Beta blocker	-3.34 (-14.57 to 7.89)	0.56
Digoxin	-2.71 (-14.63 to 9.21)	0.65
Ivabradine	12.82 (-5.30 to 30.94)	0.16
Loop diuretic	1.37 (-8.41 to 11.15)	0.78
Nitrate	-9.24 (-24.63 to 3.15)	0.14
Thiazide diuretic	-13.77 (-49.04 to 21.50)	0.44
Type of HF (HFrEF vs HFpEF)	-2.57 (-15.59 to 10.46)	0.70
ISWT (peak distance) (N=188) baseline	0.06 (0.02 to 0.09)	0.001
Overall ENMO	1.12 (-0.23 to 2.47)	0.10
MLHFQ	-0.12 (-0.32 to 0.07)	0.21
Overall		
Physical	-0.26 (-0.65 to 0.13)	0.20
Emotional	-.025 (-0.86 to 0.36)	0.42
HADS		
Anxiety	0.30 (-0.71 to 1.32)	0.56
Depression	-0.01 (-1.39 to 1.37)	0.99
HeartQoL		
Global	4.36 (-1.58 to 10.30)	0.15
Physical	4.52 (-0.97 to 10.00)	0.11
Emotional	1.56 (-3.62 to 6.75)	0.55
EQ-5D-5L (N=196)	7.54 (-10.96 to 26.03)	0.42

For peer review only

SCHFI		
Maintenance	-0.32 (-0.60 to -0.04)	0.03
Management (N=106)	0.06 (-0.22 to 0.34)	0.69
Confidence	-0.07 (-0.25 to 0.11)	0.43

BMI: body mass index; NYHA: New York Heart Association; HF: heart failure; LVEF: left ventricular ejection fraction; NT-proBNP: N-terminal proB-type natriuretic peptide; MI: myocardial infection; COPD: chronic obstructive pulmonary disease; HFrEF: heart failure with reduced ejection fraction; HFpEF: heart failure with preserved ejection fraction; ISWT: incremental shuttle walk test; ENMO: Euclidean norm minus one; MLHFQ: Minnesota living with heart failure questionnaire; HADS: hospital anxiety and depression scale; SCHFI: self-care in heart failure index.

For peer review only

Supplementary table 8: Comparison of multivariable models to predict change in minutes/day MVPA at post-intervention follow up.

Multivariable model	Variables included in model ($p<0.05$)	Unstandardized beta coefficient (95% CI)	t-statistic	Variable P-value	Model Adjusted R^2 (p-value)
1. Socio-demographic	Group	0.48 (-6.24 to 7.22)	0.14	0.89	0.10 (<0.001)
	Baseline MVPA	-0.12 (-0.18 to -0.06)	-3.81	<0.001	
	Centre	0.18 (-2.12 to 2.54)	0.15	0.88	
	BNP <>2000	-3.93 (-12.96 to 5.11)	-0.86	0.39	
	Live with parent	34.98 (13.41 to 56.55)	3.20	0.002	
	Diabetes	-12.17 (-20.08 to -4.27)	-3.04	0.003	
	constant	7.13 (-2.08 to 16.34)	1.53	0.13	
2. Exercise capacity and health status	Group	0.18 (-6.0 to 6.34)	0.06	0.95	0.10 (<0.001)
	Baseline MVPA	-0.15 (-0.21 to -0.09)	-4.73	<0.001	
	Centre	0.54 (-1.61 to 2.68)	0.49	0.62	
	BNP 2000	-2.03 (-10.29 to 6.23)	-0.49	0.63	
	ISWT peak	0.04 (0.02 to 0.07)	3.52	0.001	
	constant	-6.39 (-16.77 to 3.99)	-1.21	0.23	
3. Socio-demographic, exercise capacity and health status*	Group	2.02 (-4.8 to 8.83)	0.58	0.56	0.14 (<0.001)
	Baseline MVPA	-0.16 (-0.23 to -0.09)	-4.55	<0.001	
	Centre	0.80 (-1.59 to 3.20)	0.66	0.51	
	BNP 2000	-3.67 (-12.83 to 5.48)	-0.79	0.43	
	Live with parent	37.47 (13.69 to 61.24)	3.11	0.002	
	Diabetes	-11.99 (-19.98 to -4.01)	-2.96	0.003	
	ISWT peak	0.04 (0.01 to 0.06)	2.82	0.005	
	constant	-2.75 (-14.31 to 8.82)	-0.47	0.64	

MVPA: moderate-to-vigorous physical activity; BNP 2000: NT-proBNP above or below 2000 pg/ml; ISWT: incremental shuttle walk test; HADS: hospital anxiety and depression score
* all variables p<0.05 from multivariate models 1 and 2

For peer review only

For peer review only - http://bmjopen.bmj.com/site/about/guidelines.xhtml

Reporting checklist for randomised trial.

Based on the CONSORT guidelines.

Instructions to authors

Complete this checklist by entering the page numbers from your manuscript where readers will find each of the items listed below.

Your article may not currently address all the items on the checklist. Please modify your text to include the missing information. If you are certain that an item does not apply, please write "n/a" and provide a short explanation.

Upload your completed checklist as an extra file when you submit to a journal.

In your methods section, say that you used the CONSORTreporting guidelines, and cite them as:

Schulz KF, Altman DG, Moher D, for the CONSORT Group. CONSORT 2010 Statement: updated guidelines for reporting parallel group randomised trials

		Reporting Item	Page Number
**Title and Abstract**			
Title	#1a	Identification as a randomized trial in the title.	1
Abstract	#1b	Structured summary of trial design, methods, results, and conclusions	2-3
**Introduction**			

For peer review only

Background and objectives	#2a	Scientific background and explanation of rationale	4
Background and objectives	#2b	Specific objectives or hypothesis	4-5
**Methods**			
Trial design	#3a	Description of trial design (such as parallel, factorial) including allocation ratio.	5
Trial design	#3b	Important changes to methods after trial commencement (such as eligibility criteria), with reasons	N/A - secondary analysis, full details provided in references 12-15
Participants	#4a	Eligibility criteria for participants	6
Participants	#4b	Settings and locations where the data were collected	6-7
Interventions	#5	The experimental and control interventions for each group with sufficient details to allow replication, including how and when they were actually administered	5-6
Outcomes	#6a	Completely defined prespecified primary and secondary outcome measures, including how and when they were assessed	6-7

For peer review only

Sample size	#7a	How sample size was determined.	N/A - secondary analysis, full details provided in references 12-15
Sample size	#7b	When applicable, explanation of any interim analyses and stopping guidelines	N/A - secondary analysis, full details provided in references 12-15
Randomization - Sequence generation	#8a	Method used to generate the random allocation sequence.	
N/A - secondary analysis, full details provided in references 12-15			
Randomization - Sequence generation	#8b	Type of randomization; details of any restriction (such as blocking and block size)	
N/A - secondary analysis, full details provided in references 12-15			
Randomization - Allocation	#9	Mechanism used to implement the random allocation sequence (such as sequentially	N/A - secondary analysis, full details

For peer review only

concealment mechanism		numbered containers), describing any steps taken to conceal the sequence until interventions were assigned	provided in references 12-15
Randomization - Implementation	#10	Who generated the allocation sequence, who enrolled participants, and who assigned participants to interventions	N/A - secondary analysis, full details provided in references 12-15
Blinding	#11a	If done, who was blinded after assignment to interventions (for example, participants, care providers, those assessing outcomes) and how.	N/A - secondary analysis, full details provided in references 12-15
Blinding	#11b	If relevant, description of the similarity of interventions	N/A - secondary analysis, full details provided in references 12-15
Statistical methods	#12a	Statistical methods used to compare groups for primary and secondary outcomes	8-9
Statistical methods	#12b	Methods for additional analyses, such as subgroup analyses and adjusted analyses	8-9
Outcomes	#6b	Any changes to trial outcomes after the trial commenced, with reasons	N/A

Results

For peer review only

Participant flow diagram (strongly recommended)	#13a	For each group, the numbers of participants who were randomly assigned, received intended treatment, and were analysed for the primary outcome	Figure 1
Participant flow	#13b	For each group, losses and exclusions after randomization, together with reason	Figure 1, page 9
Recruitment	#14a	Dates defining the periods of recruitment and follow-up	6
Recruitment	#14b	Why the trial ended or was stopped	N/A - secondary analysis, full details provided in references 12-15
Baseline data	#15	A table showing baseline demographic and clinical characteristics for each group	Table 1
Numbers analysed	#16	For each group, number of participants (denominator) included in each analysis and whether the analysis was by original assigned groups	Table 2
Outcomes and estimation	#17a	For each primary and secondary outcome, results for each group, and the estimated effect size and its precision (such as 95% confidence interval)	Table 2

For peer review only

Outcomes and estimation	#17b	For binary outcomes, presentation of both absolute and relative effect sizes is recommended	Table 3
Ancillary analyses	#18	Results of any other analyses performed, including subgroup analyses and adjusted analyses, distinguishing pre-specified from exploratory	Table 4 and supplementary tables
Harms	#19	All important harms or unintended effects in each group (For specific guidance see CONSORT for harms)	N/A - secondary analysis, full details provided in references 12-15
**Discussion**			
Limitations	#20	Trial limitations, addressing sources of potential bias, imprecision, and, if relevant, multiplicity of analyses	13-14
Interpretation	#22	Interpretation consistent with results, balancing benefits and harms, and considering other relevant evidence	12-15
Registration	#23	Registration number and name of trial registry	5
Generalisability	#21	Generalisability (external validity, applicability) of the trial findings	12-15
**Other information**			

For peer review only

Interpretation	#22	Interpretation consistent with results, balancing benefits and harms, and considering other relevant evidence	12-15
Registration	#23	Registration number and name of trial registry	5
Protocol	#24	Where the full trial protocol can be accessed, if available	5 (references 12-15)
Funding	#25	Sources of funding and other support (such as supply of drugs), role of funders	15-16

Notes:

- 3b: N/A - secondary analysis, full details provided in references 12-15
- 7a: N/A - secondary analysis, full details provided in references 12-15
- 7b: N/A - secondary analysis, full details provided in references 12-15
- 8a: N/A - secondary analysis, full details provided in references 12-15
- 8b: N/A - secondary analysis, full details provided in references 12-15
- 9: N/A - secondary analysis, full details provided in references 12-15
- 10: N/A - secondary analysis, full details provided in references 12-15
- 11a: N/A - secondary analysis, full details provided in references 12-15
- 11b: N/A - secondary analysis, full details provided in references 12-15
- 13b: Figure 1, page 9

For peer review only

- 14b: N/A - secondary analysis, full details provided in references 12-15
- 18: Table 4 and supplementary tables
- 19: N/A - secondary analysis, full details provided in references 12-15
- 24: 5 (references 12-15) The CONSORT checklist is distributed under the terms of the Creative Commons Attribution License CC-BY. This checklist was completed on 25. March 2022 using https://www.goodreports.org/, a tool made by the EQUATOR Network in collaboration with Penelope.ai

For peer review only

BMJ Open

Home-based cardiac rehabilitation and physical activity in people with heart failure: a secondary analysis of the REACH-HF randomised controlled trials

Journal:	*BMJ Open*
Manuscript ID	bmjopen-2022-063284.R1
Article Type:	Original research
Date Submitted by the Author:	29-Dec-2022
Complete List of Authors:	Dibben, Grace; University of Glasgow, MRC/CSO Social and Public Health Sciences Unit; University of Exeter, College of Medicine and Health<br>Hillsdon, Melvyn; University of Exeter, Department of Public Health and Sport Sciences<br>Dalal, Hasnain; Royal Cornwall Hospitals NHS Trust, Research, Development and Innovation; University of Exeter Medical School, Primary Care<br>Tang, Lars; Slagelse Hospital, The Research Unit PROgrez, Department of Physiotherapy and Occupational Therapy; University of Southern Denmark, The Department of Regional Health Research<br>Doherty, Patrick Joseph; University of York, Department of Health Sciences<br>Taylor, Rod; University of Glasgow, MRC/CSO Social and Public Health Sciences Unit; University of Exeter, College of Medicine and Health
**Primary Subject Heading**:	Rehabilitation medicine
Secondary Subject Heading:	Cardiovascular medicine
Keywords:	Heart failure < CARDIOLOGY, REHABILITATION MEDICINE, SPORTS MEDICINE

SCHOLARONE™
Manuscripts

I, the Submitting Author has the right to grant and does grant on behalf of all authors of the Work (as defined in the below author licence), an exclusive licence and/or a non-exclusive licence for contributions from authors who are: i) UK Crown employees; ii) where BMJ has agreed a CC-BY licence shall apply, and/or iii) in accordance with the terms applicable for US Federal Government officers or employees acting as part of their official duties; on a worldwide, perpetual, irrevocable, royalty-free basis to BMJ Publishing Group Ltd ("BMJ") its licensees and where the relevant Journal is co-owned by BMJ to the co-owners of the Journal, to publish the Work in this journal and any other BMJ products and to exploit all rights, as set out in our licence.

The Submitting Author accepts and understands that any supply made under these terms is made by BMJ to the Submitting Author unless you are acting as an employee on behalf of your employer or a postgraduate student of an affiliated institution which is paying any applicable article publishing charge ("APC") for Open Access articles. Where the Submitting Author wishes to make the Work available on an Open Access basis (and intends to pay the relevant APC), the terms of reuse of such Open Access shall be governed by a Creative Commons licence – details of these licences and which Creative Commons licence will apply to this Work are set out in our licence referred to above.

Other than as permitted in any relevant BMJ Author's Self Archiving Policies, I confirm this Work has not been accepted for publication elsewhere, is not being considered for publication elsewhere and does not duplicate material already published. I confirm all authors consent to publication of this Work and authorise the granting of this licence.

For peer review only

Home-based cardiac rehabilitation and physical activity in people with heart failure: a secondary analysis of the REACH-HF randomised controlled trials

Grace O Dibben[a,b], Melvyn Hillsdon[c], Hasnain M Dalal[b,d], Lars Hermann Tang[e,f], Patrick Doherty[g], Rod S Taylor[a,b]

Author affiliations:

[a] Institute of Health and Wellbeing, University of Glasgow, Glasgow, UK.

[b] College of Medicine and Health, University of Exeter, Exeter, UK.

[c] Department of Sport and Health Sciences, University of Exeter, Exeter, UK.

[d] Royal Cornwall Hospitals NHS Trust, Truro, UK

[e] The Research Unit PROgrez, Department of Physiotherapy and Occupational Therapy, Næstved-Slagelse-Ringsted Hospitals, Denmark.

[f] The Department of Regional Health Research, University of Southern Denmark, Denmark.

[g] Department of Health Sciences, University of York, York, UK.

Corresponding author:

Grace Dibben, MRC/CSO Social and Public Health Sciences Unit, University of Glasgow, Berkeley Square, 99 Berkeley Street, Glasgow, G3 7HR

Grace.Dibben@glasgow.ac.uk

Word count: 3810

For peer review only - http://bmjopen.bmj.com/site/about/guidelines.xhtml

Abstract

Objectives: To quantify the impact of a home-based cardiac rehabilitation intervention (REACH-HF) on objectively assessed physical activity (PA) of patients with heart failure (HF) and explore the extent by which patient characteristics are associated with a change in PA.

Design: Secondary analysis of randomised controlled trial data.

Setting: Five centres in the UK.

Participants: 247 patients with HF (mean age 70.9±10.3 years; 28% female).

Interventions: REACH-HF versus usual care (control).

Primary and secondary outcome measures:

PA was assessed over 7 days via GENEActiv triaxial accelerometer at baseline (pre-randomisation), post-intervention (4 months) and final follow-up (6 to 12 months). Using HF patient specific intensity thresholds, intervention effects (REACH-HF vs control) on average min/day PA (inactivity, light PA and moderate-to-vigorous PA [MVPA]) over all days, week days and weekend days were examined using linear regression analysis. Multivariable regression was used to explore associations between baseline patient characteristics and change in PA.

Results: Although there was no difference between REACH-HF and control groups in 7-day PA levels post-intervention or at final follow-up, there was evidence of an increase in weekday MVPA (10.9 mins/day, 95%CI: -2.94 to 24.69), light PA (26.9 mins/day, 95%CI: -0.05 to 53.8) and decreased inactivity (-38.31 mins/day, 95%CI: -72.1 to -4.5) in favour of REACH-HF. Baseline factors associated with an increase in PA from baseline to final follow-

For peer review only - http://bmjopen.bmj.com/site/about/guidelines.xhtml

up were reduced MVPA, increased incremental shuttle walk test distance, increased HADS anxiety score, and living with a child >18 years ($p<0.05$).

Conclusions: Whilst participation in the REACH-HF home-based CR intervention did not increase overall weekly activity, patient's behaviour patterns appeared to change with increased weekday PA levels, and reduced inactivity. Baseline PA levels were highly predictive of PA change. Future focus should be on robust behavioural changes, improving overall levels of objectively assessed PA of people with HF.

Trial registrations: ISRCTN78539530 and ISRCTN86234930.

Abstract word count: 296

Strengths and limitations of this study

- The use of population specific intensity thresholds to derive PA intensity provides a more reliable and robust estimate of PA levels of patients with HF.
- The use of rigorous data processing and analysis methods to look beyond a single PA metric (i.e. average weekly MVPA) revealed interesting patterns in the data, however this increase in granularity causes a reduction in statistical power and future studies should consider this in sample size calculations
- As this was an exploratory study, there may be an increased risk of Type 1 errors with multiple repeated tests, and some frequency counts of variables included in multivariable models were low.

For peer review only

For peer review only - http://bmjopen.bmj.com/site/about/guidelines.xhtml

Introduction

Physical activity (PA) has numerous health benefits for heart failure (HF) patients including reduced HF mortality and HF hospitalisation, and improved quality of life.[1-3] Current PA guidelines recommend 150 mins/week moderate-to-vigorous PA (MVPA) or the equivalent of 30 mins/day on 5 or more days/week.[4-5]

Traditionally, cardiac rehabilitation (CR) interventions have prioritised increasing exercise capacity rather than PA behaviour, and evidence that CR increases PA toward recommended levels is lacking.[6] To date, only a small number of studies have assessed the impact of CR on PA in patients with HF, and few results (around 10% of individual results across 10 studies) indicated that CR positively impacts on PA, with studies mainly using self-report measures that are known to be prone to over-reporting.[6-7] Furthermore, in order to derive time spent in MVPA from accelerometers, raw data must be categorised into intensity levels using thresholds or cut-points derived from calibration studies, which have mostly been undertaken using young, healthy volunteers. Given the increased energy costs of PA in patients with HF, application of these 'generic' intensity thresholds risks misclassifying PA in this population and could make it difficult to tease out small changes in PA behaviour patterns. Because of this, it is unclear whether the lack of positive findings may be due to poor methods of PA measurement in patients with HF, or due to ineffective behaviour change within interventions. New HF specific accelerometer intensity thresholds for categorising PA intensity from raw accelerometer data have recently been developed, taking into account the lower resting metabolic rate and requirement for greater energy expenditure during PA in people with HF.[8]

Furthermore, although some studies have shown that PA levels are associated with a number of factors including age, body mass index (BMI), exercise capacity and disease severity in

For peer review only - http://bmjopen.bmj.com/site/about/guidelines.xhtml

patients with HF, these have been limited by cross-sectional design that do not measure within-person PA change.[9-11] Exploring the potential patient level characteristics at baseline (i.e., sociodemographics, exercise capacity and quality of life) that are associated with later changes in PA level could identify potential subgroups of patients, such as non-responders, who may require more intensive or personalised intervention.

Due to the wide range of health benefits associated with increased PA in patients with HF, further studies, using improved, objective and population specific PA assessment techniques are needed to understand and clarify the impact of CR on PA levels and the relationships between PA and patient level characteristics. Therefore the primary aim of this study was to assess the impact of a home-based CR programme (Rehabilitation Enablement in Chronic Heart Failure (REACH-HF)[12-15] on objectively measured PA using novel HF specific accelerometer thresholds for estimating intensity. In addition, we explored the patient level characteristics associated with a change in PA level.

Methods

Patient and Public Involvement

A key element of the REACH-HF intervention development process was the involvement of a local patient and public involvement (PPI) group consisting of 9 patients with HF and caregivers of people with HF. The PPI group co-created the REACH-HF intervention, and were involved in the recruitment process, and associated research questions and topic guides (for more details, see Greaves et al.[16]).

Study design

This secondary analysis used data pooled from two randomised controlled trials (RCTs) that randomised patients with HF 1:1 to a home-based CR intervention plus usual care (REACH-HF group) or to usual care alone (control group), stratified by site and N-terminal Brain

For peer review only - http://bmjopen.bmj.com/site/about/guidelines.xhtml

Natriuretic Peptide (NT-proBNP) (ISRCTN78539530 and ISRCTN86234930). The first was a pilot RCT of the REACH-HF intervention for patients with HF with preserved ejection fraction (HFpEF, left ventricular ejection fraction [LVEF] >45%), and the second was a multicentre RCT of the REACH-HF intervention for patients with HF with reduced ejection fraction (HfrEF, LVEF <45%). Accelerometry-measured PA was a secondary outcome of the original studies, and full details of these trials are presented elsewhere.[12-15]

In summary, REACH-HF is a theory-based, comprehensive self-management programme consisting of four core elements; (1) a HF self-help manual with a choice of two structured exercise programmes; a chair-based exercise DVD, and a progressive walking training programme (patients advised to exercise ≥3 times per week and gradually building in time/distance/walking pace); (2) a patient 'progress tracker' booklet designed to facilitate learning and record symptoms and other actions related to health care; (3) a 'family and friends resource' manual for caregivers to increase their understanding of HF and their own physical and mental wellbeing; and (4) facilitation by specially trained cardiac nurses, physiotherapists, or exercise therapists to individually tailor the intervention. Participating patients and caregivers worked through the REACH-HF manual for 12 weeks. Participants in both the pilot RCT and full RCT received the same programme.

This study complies with the Declaration of Helsinki. Ethical approval was granted for the two trials by East of Scotland Research Ethics Service (15/ES/0036) and the North West Lancaster Research Ethics Committee (14/NW/1351).

Participants

A total of 247 adults (aged ≥18 years) with a confirmed diagnosis of HF (198 HfrEF, 49 HfpEF), and complete baseline accelerometer data sets (participants were required to have ≥16 hours per day and ≥7 days of wear) were included in this analysis. Participants were

For peer review only - http://bmjopen.bmj.com/site/about/guidelines.xhtml

recruited and completed the baseline visit between January 2015 and February 2016, from primary and secondary care settings in five UK centres (Birmingham, Cornwall, Dundee, Gwent, and York). All participants provided written informed consent.

Data collection

PA data were collected via accelerometry on three occasions: at baseline (pre-randomisation), post-intervention (4 months) and final follow-up (6-12 months). Given the final accelerometry data follow-up for the two trials was at 6 months for HFpEF patients, and 12 months for HFrEF patientswe have therefore combined data at these two 'final follow-up' timepoints for this study.

The following patient level data were collected at baseline: past medical history (i.e. comorbidities, New York Heart Association (NYHA) class, concomitant medication), sociodemographic information (i.e. age, ethnicity, employment status, smoking status), NT-proBNP measurement via blood sample, exercise tolerance via incremental shuttle walk test (ISWT), and health outcome questionnaires i.e. disease-specific HRQoL using the Minnesota Living with Heart Failure Questionnaire (MLHFQ), and the Health Related Quality of Life (HeartQoL) questionnaire; psychological wellbeing using the Hospital Anxiety and Depression Scale (HADS) questionnaire; generic HRQoL using the EQ-5D-5L questionnaire; and Self-care of HF Index questionnaire (SCHFI).

Physical activity – accelerometry

At the clinic visits, participants were provided with and instructed to wear a GENEActiv triaxial accelerometer (GENEActiv, Activinsights, Kimbolton, Cambridge, UK) for 24-consecutive hours for 7 days. Accelerometers were returned to the clinical trials unit using postage-paid envelopes. Data were downloaded using GENEActiv PC software (version 3.2; Activinsights, Kimbolton, Cambridge, UK) and processed in R (R Core Team, Vienna,

For peer review only - http://bmjopen.bmj.com/site/about/guidelines.xhtml

Austria) using the GGIR software package (version 1.5-18, http://cran.r-project.org). Initial processing included autocalibration, and detection of abnormally high values and non-wear.[17-18] Data were averaged over 5 second epochs and Euclidean Norm Minus One (ENMO) was used to quantify the acceleration related to movement registered and expressed in milligravity units (mg) using the following formula.[19]

$$\sqrt{x^2 + y^2 + z^2} - 1000mg$$

Non-wear was determined over 60-minute windows using 15 minute increments, and was apparent when 2 of the 3 axes had a data range <50 mg and a SD <13 mg.[20] The first consecutive seven days that met the criteria were used for analysis.

Once the raw accelerometer data was processed, HF specific accelerometer intensity thresholds (inactivity (i.e. <1.5 metabolic equivalents [METs]: 16.7mg (left wrist), 18.6mg (right wrist), MVPA (i.e. ≥3.0METs): 43.6mg (left wrist), 45.5mg (right wrist)) were applied to calculate the average minutes per day spent inactive, in light PA and MVPA, over all days, weekend days and weekdays. These thresholds were established by a recent accelerometer calibration study in 21 patients with HF.[8] Average weekly MVPA was used to calculate the proportion of patients meeting current PA guidelines (≥150 minutes per week). For the primary analysis these metrics were calculated using bouted data, i.e., sustained periods of 10 minutes or more where accelerometer data lies above the intensity threshold (with a 20% allowance for values to fall outside the threshold). For secondary analyses, these metrics were calculated using unbouted data, i.e. allowing PA to be accumulated in bouts of any duration.

Statistical analysis

Descriptive statistics were calculated as means and standard deviations or counts and percentages unless otherwise stated.

For peer review only - http://bmjopen.bmj.com/site/about/guidelines.xhtml

Data from final follow-up were used for primary analysis in line with the primary end-points of the two original trials.[12-13] The intervention effects (i.e. REACH-HF vs control) on average min/day PA (inactivity, light PA and MVPA) over all days, week days and weekend days were examined using linear regression analysis, adjusting for baseline PA (inactivity, light PA or MVPA respectively), treatment group and trial stratification variables (NT-proBNP, centre). Secondary analyses included (i) exploring intervention effects at post-intervention follow-up; (ii) using unbouted PA data, and (iii) examining the proportion of patients meeting PA guidelines were examined using logistic regression.

Univariate linear regression was used to investigate the baseline sociodemographic (e.g. age, sex, ethnicity), medical history (e.g. NYHA class, medication), ISWT and health status variables (e.g. HRQoL) associated with change in MVPA, adjusting for baseline MVPA, treatment group and trial stratification variables (NT-proBNP, centre).

In addition to univariate regression, we undertook multivariable regression to explore the association between selected patient characteristics and change in MVPA. Variables with statistical evidence of univariate association with change in MVPA ($p<0.15$) were selected for entry into a series of multivariable regression models to establish which variables were independently and most strongly associated with change in PA at final follow-up and post-intervention, mutually adjusting for trial stratification variables, baseline MVPA and treatment group. Model 1 – sociodemographic and medical history variables only, model 2 – exercise capacity and health status variables only, and model 3 – sociodemographic, medical history, exercise capacity and health status variables identified as significant ($p<0.05$) predictors in models 1 and 2. Checks and diagnostics were performed for model assumptions, residuals, multicollinearity (variance inflation factor) and influential observations (Cook's distance). AIC and R^2 values were used to inform model comparison and selection.

For peer review only - http://bmjopen.bmj.com/site/about/guidelines.xhtml

Statistical analyses were performed using Stata (V.15.0; StataCorp, College Station, Texas, USA).

Results

247 patients had accelerometer data at baseline which met the criteria for inclusion in analysis. Post-intervention, 198 patients were included, and at final follow-up 173 patients were included in the analysis (Figure 1). Patients were predominantly male (72%), had a mean age of 70.9 ± 10.4 years, and 60% were NYHA class II (table 1), full baseline characteristics are described in supplementary table 1. At baseline, the average daily MVPA in REACH-HF group was 43.6 mins with 53% participants meeting weekly PA recommendations, and in the control group was 49.7 mins per day, with 48% participants meeting weekly PA recommendations.

Table 1: Summary baseline patient characteristics. Data are presented as N(%) unless otherwise stated.

	REACH-HF (N=122)	Control (N=125)
Mean (SD) Age (years)	70.5 (10.0)	71.3 (10.7)
Female Sex	40 (33)	30 (24)
Mean (SD) BMI (kg/m^2)	29.9 (6.6)	30.0 (5.9)
Ethnicity (white)	115 (94)	121 (97)
Type of HF (HFpEF)	24 (20)	25 (20)
NYHA class		
NYHA I	24 (20)	16 (13)
NYHA II	71 (58)	76 (61)
NYHA III-IV	27 (22)	33 (26)
Mean (SD) LVEF (%)	38.4 (14.7)	38.1 (15.5)

For peer review only - http://bmjopen.bmj.com/site/about/guidelines.xhtml

For peer review only

Mean (SD) NT-proBNP (pg/ml)	1288.3 (1794.3)	1364.4 (1602.1)
Trial site		
Truro	27 (22)	29 (23)
Gwent	22 (18)	22 (18)
Birmingham	24 (20)	24 (19)
York	25 (20)	25 (20)
Dundee	24 (20)	25 (20)
Total number of comorbidities* (median, range)	3 (2-5)	3 (2-5)
Mean (SD) ISWT (peak distance, m)	241.9 (157.1)	219.1 (144.2)
Mean (SD) MLHFQ		
Overall	33.5 (24.5)	30.7 (23.0)
Physical	17.3 (11.8)	16.1 (11.6)
Emotional	7.7 (7.7)	7.1 (7.0)
Mean (SD) HADS		
Anxiety	5.3 (4.5)	5.9 (4.4)
Depression	4.8 (3.6)	4.8 (3.4)
Mean (SD) HeartQoL		
Global	1.8 (0.8)	1.8 (0.8)
Physical	1.6 (0.8)	1.6 (0.8)
Emotional	2.1 (0.9)	2.1 (0.9)
Mean (SD) EQ-5D-5L	0.7 (0.3)	0.7 (0.3)
Mean (SD) SCHFI		
Maintenance	56.0 (16.1)	53.0 (15.4)
Management	42.0 (24.7)	39.6 (20.4)

For peer review only - http://bmjopen.bmj.com/site/about/guidelines.xhtml

For peer review only

Confidence	61.9 (25.2)	63.6 (23.4)

REACH-HF: Rehabilitation enablement in chronic heart failure; SD: standard deviation; BMI: body mass index; NYHA: New York Heart Association; HF: heart failure; LVEF: left ventricular ejection fraction; NT-proBNP: N-terminal proB-type natriuretic peptide; HFrEF: heart failure with reduced ejection fraction; HFpEF: heart failure with preserved ejection fraction; ISWT: incremental shuttle walk test; MLHFQ: Minnesota living with heart failure questionnaire; HADS: hospital anxiety and depression scale; SCHFI: self-care in heart failure index.

* Comorbidities including: angina, diabetes, myocardial infarction, hypertension, osteoporosis, stroke, asthma, chronic back pain, chronic renal impairment, arthritis, atrial fibrillation, chronic obstructive pulmonary disease, depression.

Effects of REACH-HF intervention on PA at final follow-up

The effects of REACH-HF intervention versus control at final follow-up on PA (MVPA, light PA and inactivity) over all days, weekdays and weekend days are presented in table 2. Over all 7 days, the average change in daily MVPA at final follow-up in the REACH-HF group was 4.0 mins/day, compared to -5.1 mins/day in the control group ($p>0.05$). In both groups, there was huge variance (minimum -91.2, maximum 291.7 min/day; and minimum -135.7, maximum 173.2 min/day, respectively). Over weekend days, there were no significant between group differences at any intensity, although it appeared that both groups demonstrated similar small reductions in MVPA (-5.0 and -4.6 min/day respectively), and the REACH-HF group increased their inactivity (11.8 min/day). Over weekdays, there was evidence of an increase in PA over all intensities in the REACH-HF group compared to the control group, however only the increase in light PA and decrease in inactivity reached

For peer review only

For peer review only - http://bmjopen.bmj.com/site/about/guidelines.xhtml

statistical significance (between group differences in favour of REACH-HF: light PA: 26.87 (95% CI -0.05 to 53.78), p=0.05, inactivity: -38.31 (95% CI -72.13 to -4.5), p=0.03).

For peer review only

For peer review only - http://bmjopen.bmj.com/site/about/guidelines.xhtml

Table 2: Intervention effects on PA outcomes at final follow-up

	Baseline		Final follow-up		Δ to final follow-up		Between group difference (mean, 95% CI) p-value
	REACH-HF (N=80) mean (SD)	Control (N=93) mean (SD)	REACH-HF (N=80) mean (SD)	Control (N=93) mean (SD)	REACH-HF(N=80) mean (SD)	Control (N=93) mean (SD)	
All days Bouted							
MVPA (min/day)	43.62 (51.15)	49.66 (73.98)	47.61 (67.78)	44.56 (72.10)	3.99 (51.53)	-5.10 (34.23)	7.61 (-5.11 to 20.33) p=0.24
Light (min/day)	213.80 (110.94)	219.93 (114.73)	221.92 (129.57)	209.50 (120.90)	8.12 (87.01)	-10.43 (87.85)	16.87 (-8.79 to 42.54) p=0.20
Inactive (min/day)	1182.58 (144.43)	1170.41 (164.42)	1170.47 (170.47)	1185.78 (167.52)	-12.12 (115.83)	15.53 (10.52)	-24.77 (-56.69 to 7.16) p=0.13
Weekend Days Bouted							
MVPA (min/day)	43.72 (56.62)	41.0 (69.84)	38.67 (61.04)	36.43 (62.10)	-5.05 (52.27)	-4.57 (43.31)	0.14 (-12.92 to 13.21) p=0.98

For peer review only - http://bmjopen.bmj.com/site/about/guidelines.xhtml

Light (min/day)	208.10 (123.23)	202.18 (118.25)	201.36 (128.36)	206.22 (123.41)	-6.75 (107.42)	4.04 (103.72)	-8.38 (-37.96 to 21.20) p=0.58
Inactive (min/day)	1188.18 (161.84)	1196.82 (160.52)	1199.98 (162.57)	1197.35 (158.21)	11.80 (131.33)	0.53 (121.78)	8.27 (-27.01 to 43.55) p=0.64
Week Days Bouted							
MVPA (min/day)	43.58 (50.71)	53.12 (79.66)	51.19 (71.70)	47.82 (78.90)	7.61 (55.14)	-5.31 (37.46)	10.87 (-2.94 to 24.69) p=0.12
Light (min/day)	216.08 (112.68)	227.03 (120.85)	230.15 (135.37)	210.81 (125.37)	14.07 (90.71)	-16.22 (92.74)*	26.87 (-0.05 to 53.78) p=0.05
Inactive (min/day)	1180.34 (143.59)	1159.85 (174.23)	1158.66 (178.56)	1181.37 (178.59)	-21.68 (122.78)	21.53 (106.40)*	-38.31 (-72.13 to -4.50) p=0.03

REACH-HF: Rehabilitation enablement in chronic heart failure; SD: standard deviation; MVPA: moderate-to-vigorous physical activity; PA: physical activity

* p<0.05 REACH-HF group vs control

For peer review only - http://bmjopen.bmj.com/site/about/guidelines.xhtml

Secondary analyses

(i) Effects of REACH-HF intervention on PA at post-intervention follow-up

At post-intervention follow-up, there were no significant differences between REACH-HF group and control for all bouted PA intensities or days (all, weekend or week) (supplementary table 2).

(ii) Unbouted data

At final follow-up, using unbouted PA data, there was a small increase in weekday MVPA and decrease in weekday inactivity in the REACH-HF group, with the reverse found in control group, (between group differences showing a trend in favour of REACH-HF (MVPA: 15.18 (95% CI -0.32 to 30.67), $p=0.06$; inactivity: -21.25 (95% CI -43.24 to 0.75), $p=0.06$; supplementary table 3). The REACH-HF group also appeared to increase their inactivity and decrease PA on weekend days. At post-intervention follow-up there were no significant differences between REACH-HF group and control for all bouted PA intensities or days (all, weekend or week) (supplementary table 4).

(iii) Prevalence of meeting PA guidelines

In terms of prevalence of meeting PA guidelines, no differences were found between REACH-HF and control group at either follow-up time point (supplementary tables 5 and 6).

Univariate regression analysis

Supplementary table 7 shows the univariate associations between baseline sociodemographic, clinical and behavioural patient variables and change in MVPA at final follow-up. Older patients were more likely to show a decrease in MVPA ($p<0.05$), whereas patients living with

For peer review only - http://bmjopen.bmj.com/site/about/guidelines.xhtml

a child >18 years, with greater ISWT distance, and higher HADs anxiety score were more likely to increase their MVPA ($p<0.05$).

Post-intervention, presence of diabetes and SCHFI maintenance scores at baseline were associated with a decrease in MVPA ($p<0.05$), whereas individuals with greater ISWT distance at baseline were more likely to increase their MVPA ($p<0.05$; supplementary table 8).

Multivariable regression analysis at final follow-up

Table 3 shows the multivariable prediction models at final follow-up. In model 1: higher baseline MVPA and living with a parent was associated with a decrease in MVPA and living with a child aged >18 years was associated with an increase in MVPA. This model accounted for 15% of the variance in change in MVPA. Three patients were removed with high residual (e=211.5, e=258.3 and e=182.0) and Cook's distance d=0.35, d=0.39 and d=0.04). In model 2: higher baseline MVPA was associated with a decrease in MVPA, and higher ISWT distance and HADS anxiety score were associated with an increase in MVPA. This model accounted for 9% of the variance in MVPA change. In model 3: higher ISWT distance, living with a child aged over 18 years, and higher HADS anxiety score were strongly associated with an increase in MVPA, and living with a parent and higher baseline MVPA were associated with a decrease in MVPA. The model explained 15% of the variance in MVPA change at final follow-up.

Secondary analyses

Multivariable regression analysis at post-intervention follow-up

Multivariable models to predict change in MVPA post-intervention are presented in supplementary table 9. In model 1: living with a parent was associated with an MVPA increase, and higher baseline MVPA and presence of diabetes with an MVPA decrease. This

For peer review only - http://bmjopen.bmj.com/site/about/guidelines.xhtml

model explained 10% of the variance in MVPA change. In model 2: higher baseline MVPA was associated with a decrease in MVPA, and higher ISWT distance was most strongly associated with an increase in MVPA. The model accounted for 10% of the variance in MVPA change. In model 3: living with a parent and higher ISWT distance were associated with MVPA increases, and higher baseline MVPA and presence of diabetes were strongly associated with MVPA decrease. This model accounted for 14% of the variance in MVPA change.

VIF ranged from 1.06 – 1.18 across the 6 models indicating a low level of multicollinearity.

For peer review only

For peer review only - http://bmjopen.bmj.com/site/about/guidelines.xhtml

Table 3: Comparison of multivariable models to predict change in minutes/day MVPA at final follow-up.

Multivariable model	Variables included in model ($p<0.05$)	Unstandardized beta coefficient (95% CI)	t-statistic	Variable P-value	Model Adjusted R^2 (p-value)
1. Socio-demographic	Group	-4.03 (-12.19 to 4.12)	-0.98	0.33	0.15
	Baseline MVPA	-0.15 (-0.22 to -0.09)	-4.64	<0.001	(<0.001)
	Centre	-0.28 (-3.0 to 2.45)	-0.20	0.84	
	BNP 2000	-6.58 (-17.27 to 4.10)	-1.22	0.23	
	Live with parent	-41.81 (-72.96 to -10.67)	-2.65	0.009	
	Live with child over 18	16.04 (0.05 to 32.02)	1.98	0.049	
	constant	5.89 (-4.92 to 16.70)	1.08	0.28	
2. Exercise capacity and health status	Group	-6.72 (-19.81 to 6.37)	-1.01	0.31	0.09 (0.001)
	Baseline MVPA	-0.20 (-0.31 to -0.09)	-3.47	0.001	
	Centre	0.13 (-4.34 to 4.60)	0.06	0.95	
	BNP 2000	-7.39 (-25.08 to 10.30)	-0.83	0.41	
	ISWT peak	0.07 (0.02 to 0.11)	2.69	0.008	

For peer review only - http://bmjopen.bmj.com/site/about/guidelines.xhtml

	HADs anxiety	1.90 (0.41 to 3.38)	2.52	0.013	
	constant	-12.91 (-37.03 to 11.21)	-1.06	0.29	
3. Socio-demographic, exercise capacity and health status*	Group	-8.37 (-21.13 to 4.38)	-1.3	0.20	0.15 (<0.001)
	Baseline MVPA	-0.21 (-0.33 to -0.10)	-3.73	<0.001	
	Centre	-0.11 (-4.47 to 4.25)	-0.05	0.96	
	BNP 2000	-6.21 (-23.44 to 11.01)	-0.71	0.48	
	ISWT peak	0.08 (0.03 to 0.12)	3.16	0.002	
	Live with child >18	30.48 (5.92 to 55.04)	2.45	0.02	
	HADS anxiety	1.89 (0.44 to 3.33)	2.58	0.01	
	Live with parent	-52.60 (-100.16 to -5.05)	-2.19	0.03	
	constant	-14.51 (-38.05 to 9.04)	-1.22	0.23	

MVPA: moderate-to-vigorous physical activity; BNP 2000: NT-proBNP above or below 2000 pg/ml; ISWT: incremental shuttle walk test; HADS: hospital anxiety and depression score

* all variables p<0.05 from multivariate models 1 and 2

For peer review only - http://bmjopen.bmj.com/site/about/guidelines.xhtml

Discussion

We undertook an in-depth analysis of RCT data of a CR intervention (REACH-HF) objectively assessing PA levels of patients with HF using intensity thresholds specifically developed for this population group. When PA levels were averaged over all 7 days of the week, there was no evidence that participation in REACH-HF impacted daily PA levels compared to control. As some 48% of patients in the REACH-HF trials were already meeting UK PA guidelines at baseline (based on bouted data), it may be that there was a ceiling effect, where those already physically active would have difficulty further improving upon MVPA levels, making small changes in PA difficult to pick up statistically. This has often been the case in other CR trials.[6]

However, separating weekend and weekdays revealed important patterns in the PA response. Average weekday PA levels showed a consistent trend where MVPA and light PA increased (11 min/day and 27 min/day, respectively), and inactivity decreased (-38 min/day) in the REACH-HF group compared to the control group. Over weekend days, the reverse appeared to be true, with an increase in inactivity and decrease in PA. This pattern was not evident in either group at baseline, thereforethese findings suggest that the REACH-HF participants may have compensated for increased PA during the week by being less active at the weekend, which could account for the lack of overall PA increase found. Compensation for increased exercise with increased inactivity in the subsequent days has been demonstrated in other populations such as older adults,[21-22] and overweight/obese people.[23] There are many potential reasons for this, which may or may not be volitional, including perceived fatigue, indirect effects on self-efficacy and motivation, and reduction in typical unstructured physical activity (such as activities of daily living) on non-exercise days.[24] REACH-HF participants were advised to exercise at least 3 times per week, allowing a day's rest between each session. Participants may have perceived that completion of their exercise sessions during the

For peer review only - http://bmjopen.bmj.com/site/about/guidelines.xhtml

week meant they could "reward" themselves with inactivity at the weekend, however qualitative data would be needed to shed light on the reason for this finding.

Traditionally, CR programmes have been developed with a focus on increasing exercise capacity rather than PA promotion. In its current format we have shown modest positive influences of a home-based CR programme on weekday PA levels of patients with HF. In order to achieve greater PA benefits to patients, CR programmes may need to adapt, placing stronger emphasis on increasing unstructured daily PA behaviour, or targeting both physical activity and sedentary behaviour. This could be facilitated by simplified PA messaging such as sit less, move more, every day, which might be easier for patients to understand and sustain in the longer term, leading to higher levels of total PA and reduced sedentary time, and is likely to have significant health benefits to patients with HF.[25-27] Future interventions may also consider including long-term goal setting or behaviour change coaching.

Given the large variation in PA differences from baseline up to final follow-up in MVPA, we were interested to investigate potential factors associated with a change in MVPA. To our knowledge, this is the first study to explore this. Our results showed that particular living situations of the patient were associated with PA change (living with a parent or child >18 years), which may indicate involvement of family members is associated with PA behaviour change and self-care of patients with HF.[28] Furthermore, the close relationship between exercise capacity and PA level was demonstrated in this study, where baseline MVPA and ISWT distance were the strongest and most consistent factors associated with an increase in PA levels up to 12 months follow-up. It appeared that the fitter patients at baseline were more likely to increase PA levels up to 12 months follow up, however, it is the less fit, and less active patients that will have the most to gain from increasing their overall PA. This emphasises the importance of individually tailored interventions, where identification of the

For peer review only - http://bmjopen.bmj.com/site/about/guidelines.xhtml

patient's specific needs or deficits at the start of a CR progamme would enable more personalised therapeutic intensity or focus, in order to maximise intervention efficacy.

Strengths and limitations

This study has a number of strengths. Firstly, the objective measurement of PA, and robust and rigorous accelerometer data processing and analysis. Literature reporting accelerometer measured PA in patients with HF with CR intervention is limited, especially in large, representative samples. The use of population specific intensity thresholds provides a more reliable estimate of PA levels of patients with HF, compared to application of commonly used thresholds based on healthy adults.[8] However, we acknowledge that these are based on a small heterogeneous sample of patients, and is not a perfect method. Application of a single threshold for MVPA to a population will always lead to a proportion of patients PA being misclassified due to heterogeneity in exercise tolerance. Therefore, further studies are required to find an alternative approach.

This study highlights the benefit of extracting more detailed PA data, looking beyond a single PA metric (i.e. average weekly MVPA), and considering within-week differences in PA patterns. In line with updated PA guidelines that acknowledge bouts less than 10 minutes can also be beneficial for health,[4] we also looked at the unbouted data. We found that removing the bout rule showed that 100% of the participants were meeting PA recommendations. This also provides useful information in patients with HF as performing continuous bouts of exercise for 10 minutes or more may be challenging with limited exercise tolerance. However, in increasing granularity, statistical power is reduced, and future studies should consider this in sample size calculations. Future studies could also consider the distribution of PA both between- and within-days in patients with HF, since research has shown that afternoon and evening PA decreases with increasing age.[27] This could inform future

For peer review only

intervention development, targeting inactive periods throughout both the week and the day best placed for PA modification.

As this was an exploratory study, multiple repeated independent tests were conducted comparing treatment groups, and between baseline characteristics and change in MVPA. Given the dangers of multiple testing, which may have led to increased risk of Type 1 errors, our results must be treated with caution. In addition, variables associated with MVPA change were inconsistent across the two follow-up points and explained only a small proportion (10-15%) of the variance in MVPA change and combining the final follow-up data sets from 6 and 12 months may have introduced variation to the data. Whilst the sample size was sufficient for this exploratory study, some of the frequency counts of variables included in the multivariable models were low. Further studies using objective PA assessment are needed to clarify the impact of exercise-based CR interventions in patients with HF and the patient factors associated with change in PA.

Conclusion

This pooled analysis of randomised controlled trials shows that participation in a home-based CR intervention did not impact 7-day PA compared to control. However, participating in CR did appear to increase weekday PA levels and participants may have compensated for this by becoming more inactive during the weekend. Understanding of the behavioural effects of a home-based CR intervention could provide key targets for clinicians and researchers to adapt CR focus and PA messaging, in order to encourage patients to increase overall weekly PA and reduce inactivity. Understanding the factors associated with change in PA with CR intervention could potentially enhance development of more intense interventions specifically tailored or targeted at those with lower baseline PA or exercise capacity levels, as these patients will have the most to gain from increasing their PA.

For peer review only - http://bmjopen.bmj.com/site/about/guidelines.xhtml

Acknowledgments

The authors thank the patients in the REACH-HF trials and the research team for open access to the trial data. GD completed this research whilst supported by a University of Exeter Postgraduate Studentship Grant.

Authors' Contributions: GD, RT, MH and HD were involved in the study conception and interpretation of results. GD and RT led the statistical analysis. GD drafted the manuscript and MH, HD, LT, PD and RT provided comments on the manuscript and signed off the final version.

Funding

This work was supported by a University of Exeter PhD studentship with support from Professor Taylor's NIHR Senior Investigator award. Further support was received from the Medical Research Council (grant ref: MC_UU_00022/1) and the Scottish Government Chief Scientist Office (grant ref: SPHSU16).

Competing interests:

RT, HD, PD and MH were investigators in the REACH-HF study.

Data sharing statement

This study used secondary RCT data that are available through the University of Exeter's Institutional Repository, Open Research Exeter (see https://ore.exeter.ac.uk). Access to these data is permitted but controlled through requests made via the repository to the chief investigator (Professor Taylor: r.s.taylor@exeter.ac.uk). Although use is permitted, this will be on the basis that the source of the data is acknowledged (including the funder) and it includes reference to the data set 'handle'.

Ethics approval

For peer review only

Ethical approval was granted for the two trials by East of Scotland Research Ethics Service (15/ES/0036) and the North West Lancaster Research Ethics Committee (14/NW/1351).

References

1. Lee H, Boo S, Yu J, et al. Physical functioning, physical activity, exercise self-efficacy, and quality of life among individuals with chronic heart failure in Korea: A cross-sectional descriptive study. *J Nurs Res* 2017;25(2):131-139.

2. Hedge SM, Claggett B, Shah AM, et al. Physical activity and prognosis in the TOPCAT trial (treatments of preserved cardiac function heart failure with an aldosterone antagonist). *Circulation* 2017;136:982-992.

3. Loprinzi PD. The effects of free-living physical activity on mortality after congestive heart failure diagnosis. *Int J Cardiol* 2016;203:598-599.

4. United Kingdom Chief Medical Officer. Start active, stay active. A report on physical activity for health from the four home countries' Chief Medical Officers. 2019 https://assets.publishing.service.gov.uk/government/uploads/system/uploads/attachment_data/file/832868/uk-chief-medical-officers-physical-activity-guidelines.pdf, (Accessed 28 Feb 2022).

5. Cowie A, Buckley J, Doherty P, et al. Standards and core components for cardiovascular disease prevention and rehabilitation. *Heart* 2019;105:510-515.

6. Dibben GO, Dalal HM, Taylor RS, et al. Cardiac rehabilitation and physical activity: systematic review and meta-analysis. *Heart* 2018;104:1394-1402.

7. Alharbi M, Bauman A, Neubeck L, et al. Measuring overall physical activity for cardiac rehabilitation participants: a review of the literature. *Heart Lung Circ* 2017;26(10):1008-1025.

For peer review only - http://bmjopen.bmj.com/site/about/guidelines.xhtml

8. Dibben GO, Gandhi MM, Taylor RS, et al. Physical activity assessment by accelerometry in people with heart failure. *BMC Sports Sci Med Rehabil* 2020;12:47.

9. Witham MD, Argo IS, Johnston DW, et al. Predictors of exercise capacity and everyday activity in older heart failure patients. *Eur J Heart Fail* 2006;8:203-207.

10. Dibben GO, Hillsdon M, Dalal HM, et al. Factors associated with objectively assessed physical activity levels of heart failure patients. *Journal of Clinical and Experimental Cardiology* 2020;11(4):655.

11. Snipelisky D, Kelly J, Levine JA, et al. Accelerometer-measured daily activity in heart failure with preserved ejection fraction: clinical correlates and association with standard heart failure severity indices. *Circ Heart Fail* 2017;10:e003878.

12. Taylor RS, Hayward C, Eyre V, et al. Clinical effectiveness and cost-effectiveness of the rehabilitation enablement in chronic heart failure (REACH-HF) facilitated self-care rehabilitation intervention in heart failure patients and caregivers: rationale and protocol for a multicentre randomised controlled trial. *BMJ Open* 2015;5(12):e009994.

13. Lang CC, Smith K, Wingham J, et al. A randomised controlled trial of a facilitated home-based rehabilitation intervention in patients with heart failure with preserved ejection fraction and their caregivers: the REACH-HFpEF pilot study. *BMJ Open* 2018;8(4):e019649.

14. Eyre V, Lang CC, Smith K, et al. Rehabilitation enablement in chronic heart failure – a facilitated self-care rehabilitation intervention in patients with heart failure with preserved ejection fraction (REACH-HFpEF) and their caregivers: rationale and protocol for a single-centre pilot randomised controlled trial. *BMJ Open* 2016;6(10):e012853.

For peer review only - http://bmjopen.bmj.com/site/about/guidelines.xhtml

15. Dalal HM, Taylor RS, Jolly K, et al. The effects and costs of home-based rehabilitation for heart failure with reduced ejection fraction: The REACH-HF multicentre randomized controlled trial. *Eur J Prev Cardiol* 2019;26(3):262-272.

16. Greaves CJ, Wingham J, Deighan C, et al. Optimising self-care support for people with heart failure and their caregivers: development of the Rehabilitation Enablement in Chronic Heart Failure (REACH-HF) intervention using intervention mapping. *Pilot and Feasibility Studies* 2016;2:37.

17. van Hees VT, Gorzelniak L, León EC, et al. Separating movement and gravity components in an acceleration signal and implications for the assessment of human daily physical activity. *PLOS One* 2013;8(4):e61691.

18. van Hees VT, Fang Z, Langford J, et al. Autocalibration of accelerometer data for free-living physical activity assessment using local gravity and temperature: an evaluation on four continents. *J Appl Physiol* 1985;117:738-744.

19. Bakrania K, Yates T, Rowlands AV, et al. Intensity thresholds on raw acceleration data: Euclidean norm minus one (ENMO) and mean amplitude deviation (MAD) approaches. *PLoS ONE* 2016;11(10):e0164045.

20. Sabia S, van Hees VT, Shipley MJ, et al. Association between questionnaire- and accelerometer assessed physical activity: the role of sociodemographic factors. *Am J Epidemiol* 2014; 179:781-790.

21. Goran MI, Poehlan ET. Endurance training does not enhance total energy expenditure in healthy elderly persons. *Am J Physiol* 1992;263:E950-957.

For peer review only - http://bmjopen.bmj.com/site/about/guidelines.xhtml

22. Goncin N, Linares A, Lloyd M, et al. Does sedentary time increase in older adults in the days following participation in intense exercise. *Aging Clinical and Experimental Research* 2020;32:2517-2527.

23. Kozey-Keadle S, Staudenmayer J, Libertine A, et al. Changes in sedentary time and physical activity in response to an exercise training and/or lifestyle intervention. *J Phys Act Health* 2014;11(7):1324-1333.

24. King NA, Caudwell P, Hopkins M, et al. Metabolic and behavioural compensatory responses to exercise interventions: barriers to weight loss. *Obesity* 2012;15(6):1373-1383.

25. Ekelund U, Tarp J, Steene-Johannessen J, et al. Dose-response associations between accelerometry measured physical activity and sedentary time and all cause mortality: systematic review and harmonised meta-analysis. *BMJ* 2019;366:l4570.

26. Buijs DM, Ramadi A, MacDonald K, et al. Quantity and quality of daily physical activity in older cardiac patients. *Canadian Journal of Cardiovascular Nursing* 2015;25(3):10-16.

27. Schrack JA, Zipunnikov V, Goldsmith J, et al. Assessing the "physical cliff": detailed quantification of age-related differences in daily patterns of physical activity. *J Gerontol A Biol Sci Med Sci* 2014;69(8):973-979.

28. Wingham J, Harding G, Britten N, Dalal H. Heart failure patients' attitudes, beliefs, expectations and experiences of self-management strategies: a qualitative synthesis. *Chronic Illn* 2013;10(2):135–54.

Figure Legends

Figure 1: participant flow diagram

Appendices

Supplementary materials provided in a separate document.

For peer review only - http://bmjopen.bmj.com/site/about/guidelines.xhtml

Figures

Provided separately.

For peer review only

For peer review only - http://bmjopen.bmj.com/site/about/guidelines.xhtml

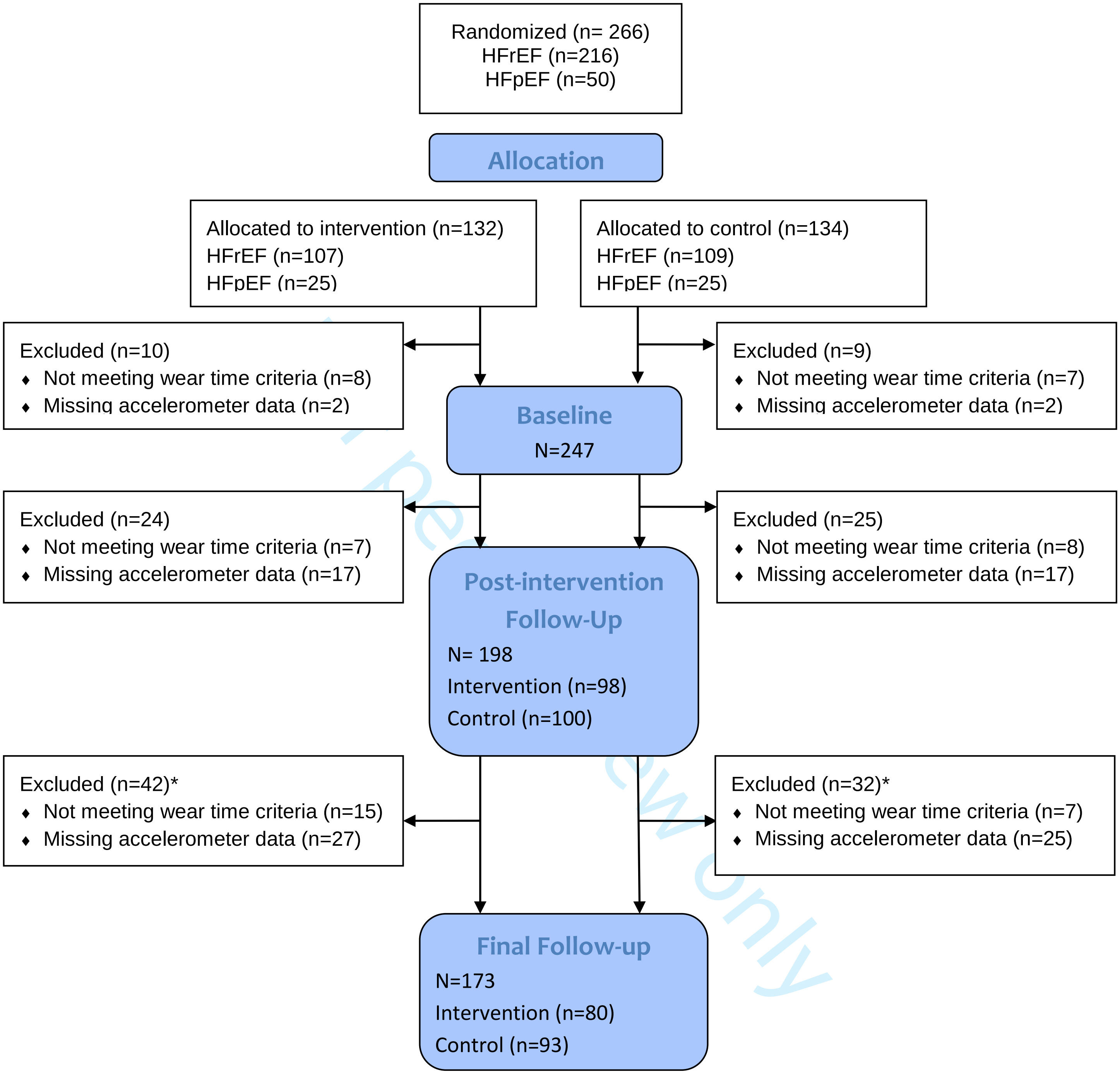

*14 patients excluded from post-intervention follow-up analysis (for missing data or not enough valid days) were included in the final follow-up with sufficient accelerometer data.

Supplementary tables

Supplementary table 1: Patient characteristics at baseline. Data are presented as N(%) unless otherwise stated.

	REACH-HF N=122	Control N=125
Mean (SD) Age (years)	70.5 (10.0)	71.3 (10.7)
Female Sex	40 (33)	30 (24)
Mean (SD) BMI (kg/m^2)	29.9 (6.6)	30.0 (5.9)
Employment status		
In employment/ Self-employed	15 (12)	16 (13)
Retired	98 (80)	101 (81)
Housework	0 (0)	1 (1)
Unemployed	7 (6)	3 (2)
Other	2 (2)	4 (3)
Ethnicity (white)	115 (94)	121 (97)
NYHA class		
NYHA I	24 (20)	16 (13)
NYHA II	71 (58)	76 (61)
NYHA III-IV	27 (22)	33 (26)
Time since HF diagnosis		
0 years	37 (30)	37 (30)
1 year	24 (20)	23 (18)
2 years	61 (50)	65 (52)
Cause of HF		
Ischaemic	52 (43)	63 (50)
Non-ischaemic	63 (52)	53 (43)
Not known/ classified	7 (5)	9 (7)
Mean (SD) LVEF (%)	38.4 (14.7)	38.1 (15.5)
Mean (SD) NT-proBNP (pg/ml)	1288.3 (1794.3)	1364.4 (1602.1)
Living alone	32 (26)	34 (27)
Living with partner	79 (65)	79 (63)
Living with child>18	7 (6)	10 (8)
Living with child<18	3 (2)	2 (2)
Living with parent	2 (2)	3 (2)
Smoking history		
Current smoker	7 (6)	7 (5)
Ex-smoker	67 (55)	72 (58)
Never smoked	48 (39)	46 (37)
Trial site		
Truro	27 (22)	29 (23)
Gwent	22 (18)	22 (18)
Birmingham	24 (20)	24 (19)
York	25 (20)	25 (20)
Dundee	24 (20)	25 (20)
Comorbidities		
Angina	32 (26)	33 (26)
Diabetes	31 (25)	28 (22)
MI	29 (24)	41 (33)
Hypertension	59 (48)	54 (43)
Osteoporosis	11 (9)	6 (5)

For peer review only

Stroke	14 (11)	15 (12)
Asthma	14 (11)	14 (11)
Chronic back pain	40 (33)	36 (29)
Chronic renal impairment	17 (14)	28 (22)
Arthritis	57 (47)	50 (40)
Atrial fibrillation	51 (42)	67 (54)
COPD	13 (11)	14 (11)
Depression	30 (25)	31 (25)
Total number of comorbidities	3 (2-5)	3 (2-5)
Medication		
Angiotensin II receptor antagonist	38 (31)	27 (22)
ACE inhibitor	71 (58)	84 (67)
Aldosterone antagonist	62 (51)	56 (45)
Anticoagulant	52 (43)	62 (50)
Beta blocker	99 (81)	95 (76)
Digoxin	23 (19)	16 (13)
Ivabradine	5 (4)	8 (6)
Loop diuretic	83 (68)	87 (70)
Nitrate	22 (18)	16 (13)
Thiazide diuretic	1 (1)	3 (2)
Type of HF (HFpEF)	24 (20)	25 (20)
Mean (SD) ISWT (peak distance, m)	241.9 (157.1)	219.1 (144.2)
Mean (SD) MLHFQ		
Overall	33.5 (24.5)	30.7 (23.0)
Physical	17.3 (11.8)	16.1 (11.6)
Emotional	7.7 (7.7)	7.1 (7.0)
Mean (SD) HADS		
Anxiety	5.3 (4.5)	5.9 (4.4)
Depression	4.8 (3.6)	4.8 (3.4)
Mean (SD) HeartQoL		
Global	1.8 (0.8)	1.8 (0.8)
Physical	1.6 (0.8)	1.6 (0.8)
Emotional	2.1 (0.9)	2.1 (0.9)
Mean (SD) EQ-5D-5L	0.7 (0.3)	0.7 (0.3)
Mean (SD) SCHFI		
Maintenance	56.0 (16.1)	53.0 (15.4)
Management	42.0 (24.7)	39.6 (20.4)
Confidence	61.9 (25.2)	63.6 (23.4)

REACH-HF: Rehabilitation enablement in chronic heart failure; SD: standard deviation; BMI: body mass index; NYHA: New York Heart Association; HF: heart failure; LVEF: left ventricular ejection fraction; NT-proBNP: N-terminal proB-type natriuretic peptide; MI: myocardial infection; COPD: chronic obstructive pulmonary disease; HFrEF: heart failure with reduced ejection fraction; HFpEF: heart failure with preserved ejection fraction; ISWT: incremental shuttle walk test; MLHFQ: Minnesota living with heart failure questionnaire; HADS: hospital anxiety and depression scale; SCHFI: self-care in heart failure index.

For peer review only

Supplementary table 2: Intervention effects on PA outcomes at post-intervention follow-up, bouted PA data

	Baseline		Post-intervention		Δ to post-intervention		Between group difference (mean, 95% CI) p-value
	REACH-HF (N=98) mean (sd)	Control (N=100) mean (sd)	REACH-HF (N=98) mean (sd)	Control (N=100) mean (sd)	REACH-HF (N=98) mean (sd)	Control (N=100) mean (sd)	
All days bouted							
MVPA (min/day)	37.90 (47.51)	44.96 (65.58)	39.64 (51.15)	46.96 (72.11)	1.74 (26.42)	2.00 (33.08)	-0.76 (-9.26 to 7.74) p=0.86
Light (min/day)	197.91 (103.22)	219.30 (108.05)	198.23 (105.26)	213.05 (114.25)	0.31 (70.48)	-6.25 (76.81)	0.98 (-19.09 to 21.06) p=0.92
Inactive (min/day)	1204.19 (133.00)	1175.74 (151.00)	1202.14 (140.26)	1179.99 (164.00)	-2.05 (76.55)	4.25 (92.03)	-2.40 (-26.20 to 21.40) p=0.84
Weekend days bouted							
MVPA (min/day)	39.01 (53.54)	37.03 (56.49)	36.62 (53.77)	43.99 (66.40)	-2.39 (33.09)	6.96 (50.56)	-9.12 (-20.82 to 2.57) p=0.13
Light (min/day)	194.02 (116.16)	198.50 (111.91)	182.82 (109.02)	192.50 (113.65)	-11.21 (88.87)	-6.00 (102.58)	-7.63 (-31.90 to 16.64) p=0.54
Inactive (min/day)	1206.97 (148.83)	1204.48 (145.62)	1220.56 (144.51)	1203.51 (155.21)	13.59 (96.46)	-0.97 (126.04)	15.84 (-13.92 to 45.59) p=0.30
Week days bouted							
MVPA (min/day)	37.46 (47.03)	48.13 (72.86)	40.85 (53.13)	48.15 (77.36)	3.39 (30.04)	0.01 (36.18)	2.26 (-7.16 to 11.67) p=0.64
Light (min/day)	199.47 (104.88)	227.63 (114.60)	204.39 (108.06)	221.27 (120.74)	4.92 (75.09)	-6.35 (81.07)	3.39 (-17.91 to 24.70) p=0.75
Inactive (min/day)	1203.07 (133.15)	1164.24 (161.83)	1194.76 (143.28)	1170.58 (174.37)	-8.31 (85.00)	6.34 (96.79)	-13.53 (-39.31 to 12.25) p=0.30

REACH-HF: Rehabilitation enablement in chronic heart failure; SD: standard deviation; MVPA: moderate-to-vigorous physical activity; PA: physical activity

For peer review only

Supplementary table 3: Intervention effects at final follow-up, unbouted PA data

	Baseline		Final follow-up		Δ to final follow-up		Between group difference (mean, 95% CI) p-value
	REACH-HF (N=80) mean (SD)	Control (N=93) mean (SD)	REACH-HF (N=80) mean (SD)	Control (N=93) mean (SD)	REACH-HF(N=80) mean (SD)	Control (N=93) mean (SD)	
All days unbouted							
MVPA (min/day)	185.98 (86.30)	190.47 (96.18)	186.76 (97.24)	180.30 (97.22)	0.78 (55.62)	-10.17 (43.89)	9.86 (-4.96 to 24.69) p=0.19
Light (min/day)	195.39 (48.24)	194.29 (46.69)	193.36 (52.48)	189.97 (46.66)	-2.03 (33.65)	-4.32 (34.66)	2.35 (-7.65 to 12.35) p=0.64
Inactive (min/day)	1058.63 (108.27)	1055.24 (118.54)	1059.88 (121.89)	1069.73 (122.72)	1.25 (73.95)	14.49 (68.52)	-11.97 (-33.12 to 9.18) p=0.27
Weekend days unbouted							
MVPA (min/day)	183.15 (91.01)	175.14 (91.13)	173.76 (94.23)	169.86 (91.67)	-9.39 (62.73)	-5.28 (54.92)	-2.87 (-19.97 to 14.23) p=0.74
Light (min/day)	193.14 (54.28)	188.28 (49.54)	185.07 (55.34)	189.01 (50.88)	-8.07 (47.07)	0.73 (45.62)	-6.97 (-19.89 to 5.94) p=0.29
Inactive (min/day)	1063.71 (177.88)	1076.58 (120.30)	1081.17 (125.01)	1081.13 (118.25)	17.46 (92.05)	4.55 (83.95)	10.07 (-15.22 to 35.35) p=0.43
Week days unbouted							
MVPA (min/day)	187.11 (86.72)	196.60 (101.66)	191.96 (100.34)	184.47 (102.63)	4.85 (58.35)	-12.13 (45.46)*	15.18 (-0.32 to 30.67) p=0.06
Light (min/day)	196.29 (48.94)	196.70 (49.06)	196.68 (53.23)	190.36 (48.31)	0.39 (33.02)	-6.34 (37.88)	6.30 (-4.05 to 16.65) p=0.23
Inactive (min/day)	1056.60 (109.35)	1046.70 (123.47)	1051.36 (124.37)	1065.17 (129.8)	-5.24 (76.14)	18.47 (72.05)*	-21.25 (-43.24 to 0.75) p=0.06

REACH-HF: Rehabilitation enablement in chronic heart failure; SD: standard deviation; MVPA: moderate-to-vigorous physical activity; PA: physical activity

* p<0.05 REACH-HF group vs control

For peer review only

Supplementary table 4: Intervention effects at post-intervention follow-up, unbouted PA data

	Baseline		Post intervention		Δ to post-intervention		Between group difference (mean, 95% CI) p-value
	REACH-HF (N=98) mean (sd)	Control (N=100) mean (sd)	REACH-HF (N=98) mean (sd)	Control (N=100) mean (sd)	REACH-HF (N=98) mean (sd)	Control (N=100) mean (sd)	
All days Unbouted							
MVPA (min/day)	171.51 (79.28)	185.77 (88.79)	170.34 (83.87)	182.84 (95.19)	-1.17 (37.95)	-2.93 (44.70)	0.58 (-11.17 to 12.34) p=0.92
Light (min/day)	191.83 (47.69)	194.95 (46.02)	189.74 (46.55)	190.66 (41.19)	-2.09 (32.45)	-4.28 (32.49)	1.14 (-7.18 to 9.46) p=0.79
Inactive (min/day)	1076.66 (102.53)	1059.28 (110.43)	1079.92 (106.13)	1066.50 (114.70)	3.27 (60.16)	7.22 (68.83)	-0.65 (-18.56 to 17.26) p=0.94
Weekend days Unbouted							
MVPA (min/day)	169.77 (84.81)	170.92 (82.42)	160.49 (86.13)	171.80 (92.20)	-9.28 (45.26)	0.88 (63.94)	-10.48 (-25.78 to 4.81) p=0.18
Light (min/day)	189.50 (53.00)	187.11 (49.14)	181.25 (52.40)	181.35 (42.84)	-8.25 (46.60)	-5.76 (42.95)	-1.75 (-12.71 to 9.20) p=0.75
Inactive (min/day)	1080.73 (113.23)	1081.98 (111.68)	1098.26 (115.25)	1086.85 (114.35)	17.53 (79.97)	4.88 (93.05)	13.10 (-9.90 to 36.11) p=0.26
Week days Unbouted							
MVPA (min/day)	172.20 (79.63)	191.72 (94.89)	174.28 (85.32)	187.26 (99.35)	2.07 (42.27)	-4.46 (46.60)	4.29 (-8.27 to 16.86) p=0.50
Light (min/day)	192.77 (48.69)	198.08 (48.26)	193.14 (46.97)	194.39 (43.64)	0.37 (33.14)	-3.69 (34.36)	2.34 (-6.30 to 10.98) p=0.59
Inactive (min/day)	1075.03 (103.46)	1050.20 (115.91)	1072.59 (106.80)	1058.36 (119.56)	-2.44 (64.34)	8.15 (71.34)	-5.43 (-24.22 to 13.36) p=0.57

REACH-HF: Rehabilitation enablement in chronic heart failure; SD: standard deviation; MVPA: moderate-to-vigorous physical activity; PA: physical activity

For peer review only

Supplementary table 5: Intervention effects on proportion of patients meeting PA guidelines at final-follow up

	Baseline		Final follow-up		OR (95% CI) p-value
	REACH-HF (N=80) n (%)	Control (N=93) n (%)	REACH-HF (N=80) n (%)	Control (N=93) n (%)	
Bouted					
Proportion meeting guidelines	42 (53%)	45 (48%)	43 (54%)	39 (42%)	0.43 (0.16 to 1.14) p=0.09
Unbouted					
Proportion meeting guidelines	80 (100%)	93 (100%)	79 (99%)	93 (100%)	-

REACH-HF: Rehabilitation enablement in chronic heart failure; OR: odds ratio

Supplementary table 6: Intervention effects on proportion of patients meeting PA guidelines at post-intervention follow-up

	Baseline		Post-intervention		OR (95% CI) p-value
	REACH-HF (N=98) n (%)	Control (N=100) n (%)	REACH-HF (N=98) n (%)	Control (N=100) n (%)	
Bouted Proportion meeting guidelines	47, (48%)	47 (47%)	49 (50%)	47 (47%)	0.79 (0.34 to 1.84) p=0.59
Unbouted Proportion meeting guidelines	98 (100%)	100 (100%)	96, (98%)	100 (100%)	-

REACH-HF: Rehabilitation enablement in chronic heart failure; OR: odds ratio

For peer review only

Supplementary table 7: Univariate association with change in MVPA at final follow-up, controlling for trial stratifiers, group and baseline MVPA

Sociodemographic, exercise capacity and health status variables<br>N=173 unless otherwise stated	Unstandardized beta coefficient (95% CI)	p-value
Age	-0.77 (-1.49 to -0.05)	0.04
Gender	1.19 (-13.50 to 15.89)	0.87
BMI	-0.46 (-1.68 to 0.75)	0.45
Employment status		
In employment/Self-employed	Comparison group	0.26
Retired	-17.13 (-38.46 to 4.19)	
Housework	-18.44 (-105.09 to 68.21)	
Unemployed	-39.19 (-82.28 to 4.41)	
Other	-38.71 (-84.28 to 6.86)	
Ethnicity (white vs other)	-20.33 (-56.51 to 15.86)	0.27
NYHA class		
NYHA I	Comparison group	0.10
NYHA II	-17.20 (-34.54 to 0.14)	
NYHA III-IV	-21.84 (-43.34 to -0.34)	
Time since HF diagnosis		
0 years	Comparison group	0.27
1 year	-11.12 (-30.01 to 7.76)	
2 years	-11.72 (-26.48 to 3.03)	
Cause of HF		
Ischaemic	Comparison group	0.44
Non-ischaemic	8.53 (-4.84 to 21.90)	
Not known/classified	0.91 (-30.46 to 32.29)	
LVEF (%) (N=137)	-0.52 (-1.57 to 0.52)	0.33
NT-proBNP (pg/ml)	-0.003 (-0.009 to 0.004)	0.42
Living alone	-3.06 (-18.40 to 12.28)	0.69
Living with partner	-3.06 (-18.40 to 12.28)	0.69
Living with child >18	25.98 (1.80 to 50.16)	0.04
Living with child <18	-1.16 (-50.64 to 48.33)	0.96
Living with parent	-45.62 (-94.86 to 3.62)	0.07
Smoking history		
Current smoker	Comparison group	0.61
Ex-smoker	16.14 (-16.57 to 48.84)	
Never smoked	16.28 (-17.23 to 49.80)	
Trial site		
Truro	Comparison group	0.17
Gwent	-9.09 (-27.90 to 9.72)	
Birmingham	15.20 (-5.94 to 36.35)	
York	-2.93 (-21.72 to 15.87)	
Dundee	-10.67 (-29.41 to 8.07)	

For peer review only

Comorbidities		
Angina	0.16 (-15.50 to 15.82)	0.98
Diabetes	-12.85 (-27.80 to 2.09)	0.09
MI	-5.75 (-20.12 to 8.62)	0.43
Hypertension	-1.92 (-15.17 to 11.33)	0.78
Osteoporosis	6.64 (-19.25 to 32.52)	0.61
Stroke	-7.21 (-27.07 to 12.64)	0.47
Asthma	4.16 (-18.08 to 26.41)	0.71
Chronic back pain	1.60 (-11.91 to 15.11)	0.82
Chronic renal impairment	-3.58 (-21.05 to 13.89)	0.69
Arthritis	9.77 (-3.38 to 22.91)	0.14
Atrial fibrillation	-10.54 (-23.55 to 2.47)	0.11
COPD	-15.62 (-35.03 to 3.79)	0.11
Depression	-2.0 (-17.05 to 13.06)	0.79
Total number of comorbidities	-1.29 (-4.45 to 1.87)	0.42
Total number of cardiorespiratory and metabolic comorbidities*	-4.56 (-9.47 to 0.35)	0.07
Total number of physical and musculoskeletal comorbidities†	3.85 (-3.25 to 10.95)	0.29
Medication		
Angiotensin II receptor antagonist	4.66 (-9.91 to 19.23)	0.53
ACE inhibitor	-0.59 (-14.16 to 12.98)	0.93
Aldosterone antagonist	4.13 (-9.52 to 17.78)	0.55
Anticoagulant	-7.62 (-20.70 to 5.47)	0.25
Beta blocker	-7.79 (-24.33 to 8.75)	0.35
Digoxin	-0.52 (-18.20 to 17.16)	0.95
Ivabradine	5.48 (-25.45 to 36.42)	0.73
Loop diuretic	-12.65 (-26.68 to 1.39)	0.08
Nitrate	-1.50 (-21.28 to 18.27)	0.88
Thiazide diuretic	-15.84 (-65.32 to 33.65)	0.53
Type of HF (HFrEF vs HFpEF)	collinearity	
ISWT (peak distance) (N=165) baseline	0.06 (0.01 to 0.12)	0.02
Overall ENMO	1.44 (-0.52 to 3.39)	0.15
MLHFQ	0.04 (-0.26 to 0.33)	0.81
Overall		
Physical	-0.14 (-0.74 to 0.47)	0.66
Emotional	0.47 (-0.43 to 1.36)	0.31
HADS		
Anxiety	1.83 (0.40 to 3.25)	0.01
Depression	-0.13 (-2.27 to 2.01)	0.91
HeartQoL		
Global	-2.09 (-11.01 to 6.82)	0.64
Physical	-0.66 (-9.02 to 7.69)	0.88
Emotional	-3.82 (-11.27 to 3.64)	0.31
EQ-5D-5L (N=172)	4.58 (-22.35 to 31.52)	0.74

For peer review only

SCHFI		
Maintenance	0.19 (-0.25 to 0.64)	0.39
Management (N=94)	-0.01 (-0.28 to 0.25)	0.92
Confidence	0.01 (-0.26 to 0.27)	0.96

BMI: body mass index; NYHA: New York Heart Association; HF: heart failure; LVEF: left ventricular ejection fraction; NT-proBNP: N-terminal proB-type natriuretic peptide; MI: myocardial infection; COPD: chronic obstructive pulmonary disease; HFrEF: heart failure with reduced ejection fraction; HFpEF: heart failure with preserved ejection fraction; ISWT: incremental shuttle walk test; ENMO: Euclidean norm minus one; MLHFQ: Minnesota living with heart failure questionnaire; HADS: hospital anxiety and depression scale; SCHFI: self-care in heart failure index.

For peer review only

Supplementary table 8: Univariate association with change in MVPA at post-intervention follow-up, controlling for trial stratifiers, group and baseline MVPA

Sociodemographic, exercise capacity and health status variables<br>N=198 unless otherwise stated	Unstandardized beta coefficient (95% CI)	p-value
Age	-0.39 (-0.85 to 0.08)	0.11
Gender	-5.74 (-15.85 to 4.37)	0.26
BMI	-0.19 (-1.00 to 0.63)	0.65
Employment status		
In employment/Self-employed	Comparison group	0.30
Retired	-10.83 (-25.08 to 3.41)	
Housework	-10.65 (-72.68 to 51.38)	
Unemployed	-29.16 (-56.92 to -1.40)	
Other	-3.58 (-32.96 to 25.81)	
Ethnicity (white vs other)	-1.91 (-27.60 to 23.77)	0.88
NYHA class		
NYHA I	Comparison group	0.17
NYHA II	-5.67 (-17.94 to 6.59)	
NYHA III-IV	-13.58 (-28.25 to 1.08)	
Time since HF diagnosis		
0 years	Comparison group	0.80
1 year	-3.15 (-16.06 to 9.76)	
2 years	-3.30 (-13.30 to 6.70)	
Cause of HF		
Ischaemic	Comparison group	0.78
Non-ischaemic	-2.90 (-11.93 to 6.12)	
Not known/classified	1.62 (-18.58 to 21.82)	
LVEF (%) (N=151)	-0.08 (-0.67 to 0.51)	0.79
NT-proBNP (pg/ml)	-0.0003 (-0.004 to 0.003)	0.87
Living alone	-5.25 (-15.76 to 5.25)	0.33
Living with partner	2.94 (-6.55 to 12.42)	0.61
Living with child >18	2.94 (-14.88 to 20.76)	0.33
Living with child <18	5.81 (-29.66 to 41.29)	0.32
Living with parent	25.68 (-1.81 to 53.16)	0.07
Smoking history		
Current smoker	Comparison group	0.21
Ex-smoker	6.47 (-14.09 to 27.04)	
Never smoked	-1.55 (-22.93 to 19.83)	
Trial site		
Truro	Comparison group	0.76
Gwent	4.19 (-8.66 to 17.03)	
Birmingham	5.20 (-8.71 to 19.12)	
York	-1.81 (-14.46 to 10.84)	
Dundee	-2.57 (-15.59 to 10.46)	

For peer review only

Comorbidities		
Angina	-1.72 (-11.93 to 8.48)	0.74
Diabetes	-12.67 (-22.52 to 2.81)	0.01
MI	3.43 (-6.40 to 12.26)	0.49
Hypertension	-2.67 (-11.57 to 6.23)	0.55
Osteoporosis	-10.64 (-29.90 to 8.61)	0.28
Stroke	-1.65 (-15.44 to 12.14)	0.81
Asthma	8.83 (-5.38 to 23.04)	0.22
Chronic back pain	-8.46 (-17.87 to 0.94)	0.08
Chronic renal impairment	0.01 (-0.05 to 0.07)	0.68
Arthritis	-2.62 (-11.67 to 6.43)	0.57
Atrial fibrillation	-2.47 (-11.36 to 6.42)	0.58
COPD	-4.18 (-18.58 to 10.21)	0.57
Depression	-3.33 (-13.99 to 7.32)	0.54
Total number of comorbidities	-1.05 (-3.13 to 1.04)	0.32
Total number of cardiorespiratory and metabolic comorbidities*	-1.77 (-5.04 to 1.51)	0.29
Total number of physical and musculoskeletal comorbidities†	-3.33 (-8.37 to 1.71)	0.19
Medication		
Angiotensin II receptor antagonist	-0.99 (-10.74 to 8.76)	0.84
ACE inhibitor	5.54 (-3.47 to 14.56)	0.23
Aldosterone antagonist	3.87 (-5.29 to 13. 03)	0.83
Anticoagulant	-8.31 (-17.13 to 0.51)	0.07
Beta blocker	-3.34 (-14.57 to 7.89)	0.56
Digoxin	-2.71 (-14.63 to 9.21)	0.65
Ivabradine	12.82 (-5.30 to 30.94)	0.16
Loop diuretic	1.37 (-8.41 to 11.15)	0.78
Nitrate	-9.24 (-24.63 to 3.15)	0.14
Thiazide diuretic	-13.77 (-49.04 to 21.50)	0.44
Type of HF (HFrEF vs HFpEF)	-2.57 (-15.59 to 10.46)	0.70
ISWT (peak distance) (N=188) baseline	0.06 (0.02 to 0.09)	0.001
Overall ENMO	1.12 (-0.23 to 2.47)	0.10
MLHFQ	-0.12 (-0.32 to 0.07)	0.21
Overall		
Physical	-0.26 (-0.65 to 0.13)	0.20
Emotional	-.025 (-0.86 to 0.36)	0.42
HADS		
Anxiety	0.30 (-0.71 to 1.32)	0.56
Depression	-0.01 (-1.39 to 1.37)	0.99
HeartQoL		
Global	4.36 (-1.58 to 10.30)	0.15
Physical	4.52 (-0.97 to 10.00)	0.11
Emotional	1.56 (-3.62 to 6.75)	0.55
EQ-5D-5L (N=196)	7.54 (-10.96 to 26.03)	0.42

For peer review only

SCHFI		
Maintenance	-0.32 (-0.60 to -0.04)	0.03
Management (N=106)	0.06 (-0.22 to 0.34)	0.69
Confidence	-0.07 (-0.25 to 0.11)	0.43

BMI: body mass index; NYHA: New York Heart Association; HF: heart failure; LVEF: left ventricular ejection fraction; NT-proBNP: N-terminal proB-type natriuretic peptide; MI: myocardial infection; COPD: chronic obstructive pulmonary disease; HFrEF: heart failure with reduced ejection fraction; HFpEF: heart failure with preserved ejection fraction; ISWT: incremental shuttle walk test; ENMO: Euclidean norm minus one; MLHFQ: Minnesota living with heart failure questionnaire; HADS: hospital anxiety and depression scale; SCHFI: self-care in heart failure index.

For peer review only

Supplementary table 9: Comparison of multivariable models to predict change in minutes/day MVPA at post-intervention follow up.

Multivariable model	Variables included in model ($p<0.05$)	Unstandardized beta coefficient (95% CI)	t-statistic	Variable P-value	Model Adjusted R^2 (p-value)
1. Socio-demographic	Group	0.48 (-6.24 to 7.22)	0.14	0.89	0.10 (<0.001)
	Baseline MVPA	-0.12 (-0.18 to -0.06)	-3.81	<0.001	
	Centre	0.18 (-2.12 to 2.54)	0.15	0.88	
	BNP <>2000	-3.93 (-12.96 to 5.11)	-0.86	0.39	
	Live with parent	34.98 (13.41 to 56.55)	3.20	0.002	
	Diabetes	-12.17 (-20.08 to -4.27)	-3.04	0.003	
	constant	7.13 (-2.08 to 16.34)	1.53	0.13	
2. Exercise capacity and health status	Group	0.18 (-6.0 to 6.34)	0.06	0.95	0.10 (<0.001)
	Baseline MVPA	-0.15 (-0.21 to -0.09)	-4.73	<0.001	
	Centre	0.54 (-1.61 to 2.68)	0.49	0.62	
	BNP 2000	-2.03 (-10.29 to 6.23)	-0.49	0.63	
	ISWT peak	0.04 (0.02 to 0.07)	3.52	0.001	
	constant	-6.39 (-16.77 to 3.99)	-1.21	0.23	
3. Socio-demographic, exercise capacity and health status*	Group	2.02 (-4.8 to 8.83)	0.58	0.56	0.14 (<0.001)
	Baseline MVPA	-0.16 (-0.23 to -0.09)	-4.55	<0.001	
	Centre	0.80 (-1.59 to 3.20)	0.66	0.51	
	BNP 2000	-3.67 (-12.83 to 5.48)	-0.79	0.43	
	Live with parent	37.47 (13.69 to 61.24)	3.11	0.002	
	Diabetes	-11.99 (-19.98 to -4.01)	-2.96	0.003	
	ISWT peak	0.04 (0.01 to 0.06)	2.82	0.005	
	constant	-2.75 (-14.31 to 8.82)	-0.47	0.64	

MVPA: moderate-to-vigorous physical activity; BNP 2000: NT-proBNP above or below 2000 pg/ml; ISWT: incremental shuttle walk test; HADS: hospital anxiety and depression score
* all variables $p<0.05$ from multivariate models 1 and 2

For peer review only

Reporting checklist for randomised trial.

Based on the CONSORT guidelines.

Instructions to authors

Complete this checklist by entering the page numbers from your manuscript where readers will find each of the items listed below.

Your article may not currently address all the items on the checklist. Please modify your text to include the missing information. If you are certain that an item does not apply, please write "n/a" and provide a short explanation.

Upload your completed checklist as an extra file when you submit to a journal.

In your methods section, say that you used the CONSORTreporting guidelines, and cite them as:

Schulz KF, Altman DG, Moher D, for the CONSORT Group. CONSORT 2010 Statement: updated guidelines for reporting parallel group randomised trials

		Reporting Item	Page Number
**Title and Abstract**			
Title	#1a	Identification as a randomized trial in the title.	1
Abstract	#1b	Structured summary of trial design, methods, results, and conclusions	2-3
**Introduction**			

For peer review only

Background and objectives	#2a	Scientific background and explanation of rationale	4
Background and objectives	#2b	Specific objectives or hypothesis	4-5
**Methods**			
Trial design	#3a	Description of trial design (such as parallel, factorial) including allocation ratio.	5
Trial design	#3b	Important changes to methods after trial commencement (such as eligibility criteria), with reasons	N/A - secondary analysis, full details provided in references 12-15
Participants	#4a	Eligibility criteria for participants	6
Participants	#4b	Settings and locations where the data were collected	6-7
Interventions	#5	The experimental and control interventions for each group with sufficient details to allow replication, including how and when they were actually administered	5-6
Outcomes	#6a	Completely defined prespecified primary and secondary outcome measures, including how and when they were assessed	6-7

For peer review only

Sample size	#7a	How sample size was determined.	N/A - secondary analysis, full details provided in references 12-15
Sample size	#7b	When applicable, explanation of any interim analyses and stopping guidelines	N/A - secondary analysis, full details provided in references 12-15
Randomization - Sequence generation	#8a	Method used to generate the random allocation sequence.	
N/A - secondary analysis, full details provided in references 12-15			
Randomization - Sequence generation	#8b	Type of randomization; details of any restriction (such as blocking and block size)	
N/A - secondary analysis, full details provided in references 12-15			
Randomization - Allocation	#9	Mechanism used to implement the random allocation sequence (such as sequentially	N/A - secondary analysis, full details

For peer review only

concealment mechanism		numbered containers), describing any steps taken to conceal the sequence until interventions were assigned	provided in references 12-15
Randomization - Implementation	#10	Who generated the allocation sequence, who enrolled participants, and who assigned participants to interventions	N/A - secondary analysis, full details provided in references 12-15
Blinding	#11a	If done, who was blinded after assignment to interventions (for example, participants, care providers, those assessing outcomes) and how.	N/A - secondary analysis, full details provided in references 12-15
Blinding	#11b	If relevant, description of the similarity of interventions	N/A - secondary analysis, full details provided in references 12-15
Statistical methods	#12a	Statistical methods used to compare groups for primary and secondary outcomes	8-9
Statistical methods	#12b	Methods for additional analyses, such as subgroup analyses and adjusted analyses	8-9
Outcomes	#6b	Any changes to trial outcomes after the trial commenced, with reasons	N/A

Results

Participant flow diagram (strongly recommended)	#13a	For each group, the numbers of participants who were randomly assigned, received intended treatment, and were analysed for the primary outcome	Figure 1
Participant flow	#13b	For each group, losses and exclusions after randomization, together with reason	Figure 1, page 9
Recruitment	#14a	Dates defining the periods of recruitment and follow-up	6
Recruitment	#14b	Why the trial ended or was stopped	N/A - secondary analysis, full details provided in references 12-15
Baseline data	#15	A table showing baseline demographic and clinical characteristics for each group	Table 1
Numbers analysed	#16	For each group, number of participants (denominator) included in each analysis and whether the analysis was by original assigned groups	Table 2
Outcomes and estimation	#17a	For each primary and secondary outcome, results for each group, and the estimated effect size and its precision (such as 95% confidence interval)	Table 2

For peer review only

Outcomes and estimation	#17b	For binary outcomes, presentation of both absolute and relative effect sizes is recommended	Table 3
Ancillary analyses	#18	Results of any other analyses performed, including subgroup analyses and adjusted analyses, distinguishing pre-specified from exploratory	Table 4 and supplementary tables
Harms	#19	All important harms or unintended effects in each group (For specific guidance see CONSORT for harms)	N/A - secondary analysis, full details provided in references 12-15
**Discussion**			
Limitations	#20	Trial limitations, addressing sources of potential bias, imprecision, and, if relevant, multiplicity of analyses	13-14
Interpretation	#22	Interpretation consistent with results, balancing benefits and harms, and considering other relevant evidence	12-15
Registration	#23	Registration number and name of trial registry	5
Generalisability	#21	Generalisability (external validity, applicability) of the trial findings	12-15
**Other information**			

For peer review only

Interpretation	#22	Interpretation consistent with results, balancing benefits and harms, and considering other relevant evidence	12-15
Registration	#23	Registration number and name of trial registry	5
Protocol	#24	Where the full trial protocol can be accessed, if available	5 (references 12-15)
Funding	#25	Sources of funding and other support (such as supply of drugs), role of funders	15-16

Notes:

- 3b: N/A - secondary analysis, full details provided in references 12-15
- 7a: N/A - secondary analysis, full details provided in references 12-15
- 7b: N/A - secondary analysis, full details provided in references 12-15
- 8a: N/A - secondary analysis, full details provided in references 12-15
- 8b: N/A - secondary analysis, full details provided in references 12-15
- 9: N/A - secondary analysis, full details provided in references 12-15
- 10: N/A - secondary analysis, full details provided in references 12-15
- 11a: N/A - secondary analysis, full details provided in references 12-15
- 11b: N/A - secondary analysis, full details provided in references 12-15
- 13b: Figure 1, page 9

For peer review only

- 14b: N/A - secondary analysis, full details provided in references 12-15
- 18: Table 4 and supplementary tables
- 19: N/A - secondary analysis, full details provided in references 12-15
- 24: 5 (references 12-15) The CONSORT checklist is distributed under the terms of the Creative Commons Attribution License CC-BY. This checklist was completed on 25. March 2022 using https://www.goodreports.org/, a tool made by the EQUATOR Network in collaboration with Penelope.ai

For peer review only
